# Genome-Wide Association Study of Natural Variation in *Arabidopsis* Exposed to Acid Mine Drainage Toxicity and Validation of Associated Genes with Reverse Genetics

**DOI:** 10.3390/plants10020191

**Published:** 2021-01-20

**Authors:** Bandana Ghimire, Thangasamy Saminathan, Abiodun Bodunrin, Venkata Lakshmi Abburi, Arjun Ojha Kshetry, Suhas Shinde, Padma Nimmakayala, Umesh K. Reddy

**Affiliations:** Gus R. Douglass Institute and Department of Biology, West Virginia State University, Institute, WV 25112-1000, USA; bghimire@wvstateu.edu (B.G.); tsaminathan@wvstateu.edu (T.S.); abodunrin@wvstateu.edu (A.B.); vabburi@wvstateu.edu (V.L.A.); aojhakshetry@wvstateu.edu (A.O.K.); suhas.shinde@wvstateu.edu (S.S.); padma@wvstateu.edu (P.N.)

**Keywords:** acid mine drainage, low pH, GWAS, natural variation, *Arabidopsis*

## Abstract

Acid mine drainage (AMD) is a huge environmental problem in mountain-top mining regions worldwide, including the Appalachian Mountains in the United States. This study applied a genome-wide association study (GWAS) to uncover genomic loci in *Arabidopsis* associated with tolerance to AMD toxicity. We characterized five major root phenotypes—cumulative root length, average root diameter, root surface area, root volume, and primary root length—in 180 *Arabidopsis* accessions in response to AMD-supplemented growth medium. GWAS of natural variation in the panel revealed genes associated with tolerance to an acidic environment. Most of these genes were transcription factors, anion/cation transporters, metal transporters, and unknown proteins. Two T-DNA insertion mutants, *At1g63005* (*miR399b*) and *At2g05635* (*DEAD helicase RAD3*), showed enhanced acidity tolerance. Our GWAS and the reverse genetic approach revealed genes involved in conferring tolerance to coal AMD. Our results indicated that proton resistance in hydroponic conditions could be an important index to improve plant growth in acidic soil, at least in acid-sensitive plant species.

## 1. Introduction

Soil contamination is a major global environmental issue, severely affecting crop production worldwide, along with bio-accumulation of toxic chemicals in our food. Anthropogenic activities are the major source of soil contamination [1]. Coal mining is the dominant driver of land-use change in the Appalachian region [2]. Coal mining has been performed industrially in the Appalachian Mountains since the mid-1800s. Today, it continues to serve as a significant contributor to the regional economy and the United States’ energy portfolio Administration [3]. West Virginia is the second-largest coal-producing state in the United States after Wyoming (https://www.eia.gov). Coal is mined in West Virginia and the other Appalachian Mountains by both underground mining and mountain-top removal methods. Mountain-top mining is a form of surface mining that involves removing the topsoil layer and exposing the coal seams. The extensive mountain-top mining and coal processing activities in Appalachia and across the world have left a legacy of contaminated mine spoils.

The discharge from active or abandoned mines is called acid mine drainage (AMD) and alters microbial communities and vegetation [4,5]. The AMD water is usually acidic and contains a high concentration of metals such as cadmium (Cd), lead (Pb), aluminum (Al), iron (Fe), selenium (Se), and manganese (Mn). These significant and uncontrolled fluxes of elements pose great threats to both ecosystems and human health in local and regional environments surrounding the mine sites. The metal sulfides within the surrounding rock and overburden become oxidized on exposure to air and water, resulting in highly acidic leachates [4] that contain toxic pollutants [6].

The major stresses phosphate (P) deficiency, high levels of Al, and proton rhizotoxicity (low pH) limit plant growth in acid soils [7]. The acidity of coal AMD leads to increased solubility of heavy metals in the soil such as Al, Fe, and Mn, which are essential components for plant growth and development. However, high concentrations of these ions interfere with critical enzymatic activities, which has deleterious effects on plants [8]. Hydrogen ions and metal cations replace essential plant cations at cation exchange sites, thus resulting in the leaching of essential elements from soil [9]. The excess concentrations also stimulate the formation of free radicals and reactive oxygen species (ROS), resulting in oxidative stress and consequently damaging the cells. Abandoned mine sites often accelerate the AMD generation process, and reclamation may require decades of proper management practices. Extensively acidic pH (as low as 2–4 standard units) coupled with metal toxicity can have severe impacts on aquatic biodiversity [10,11].

Plants have evolved a complex network of homeostatic mechanisms for resistance to toxic ion accumulation. These mechanisms include reducing uptake or efflux pump of metal ions at the plasma membrane, chelation of metals in the cytosol, and compartmentalization in vacuoles by tonoplast-located transporters. Also, the repair of stress-damaged proteins by heat shock protein or metallothionein prevents the accumulation of toxic levels of ions at sensitive sites, helping plants tolerate the deleterious effects of metal ions [12]. Plants that can grow on acidic soils have evolved mechanisms to tolerate ion toxicity stress [13,14,15,16]. Also, plants primarily respond to oxidative damage by generating ROS such as hydrogen peroxide, hydroxide free radicals, and superoxide, which affect plant physiological and biochemical processes [17]. Soil acidity also weakens the calcium-mediated cross-linkage of pectin involved in cell elongation, thereby affecting root elongation [9].

On exposure to Appalachian mine-site soil, plants showed increased expression of metal transporters, receptor kinase, chelating proteins, glutathione-s-transferase (GST), ferric reductase, channel proteins, and several signaling pathway genes [18]. This finding suggests that several genes play critical roles in plant tolerance to metal toxicity. Of note, the molecular mechanisms underlying metal toxicity and acidity tolerance are highly interrelated. Root growth inhibition is the most common effect of metal ion toxicity in plants. Al toxicity is one of the principal factors that limit plant growth in acid soils, causing root thickening, stubbiness, and distortion by affecting root cell division, thereby affecting root elongation [19,20,21]. Acidity can further disturb stress-signaling pathways, including the regulation of cytosolic free Ca^2+^ activities [22]. Many forests, invasive, and grassland species naturally adapted to acid soils, tolerate H^+^ and Al^3+,^ and thrive in soils with pH < 4.0. However, most crop plants show inhibitory growth effects under such conditions [23]. Organic acid or anion transporters genes show high expression in *Arabidopsis* seedlings exposed to acidity stress [24].

Soil acidity can damage root membranes and activate rapid H^+^ influx, depolarizing the plasma membrane and leading to cytoplasmic acidification, thus resulting in plant growth inhibition [16,25,26]. Plants that survive under Al stress appear to stimulate P uptake to alter the balance of cation–anions, enabling survival in Al toxic acid soils [27,28,29]. Common mechanisms of Al tolerance are the exudation of organic acid by activating anion channels and forming stable complexes of citrate, oxalate, and malate with Al to protect root apex [30,31,32]. Some plant species are more tolerant to Al than others [33,34,35,36]. However, the molecular mechanisms and associated genes that confer AMD toxicity tolerance remain little known.

*Arabidopsis*, used as a model organism for understanding the genetics and molecular biology of plants, has a broad natural distribution throughout Europe, Asia, and North America [37,38]. Differences in natural accessions of *Arabidopsis* are being used to uncover the complex genetic interactions underlying plant responses to the environment and the evolution of morphological traits. Genome-wide association study (GWAS) represents a promising way to study complex traits that result from a plethora of genetic variations in a wide range of plant traits. However, the study of natural variation in the adaptation to proton and metal ion toxicity of AMD is lacking. In the present study, we phenotyped five major root traits in 180 *Arabidopsis thaliana* accessions in response to AMD-supplemented growth conditions. These data were used in a GWAS approach to identify candidate genes likely involved in adaptation to AMD toxicity.

## 2. Results

### 2.1. AMD Analysis and Hydroponic Growth System

The metal constituents and pH conditions of nutrient solution, AMD, and diluted AMD are given in Table 1. Our hydroponic growth system used nutrient solution as control and diluted AMD as an experiment throughout the study. The pH, a direct measure of acidity, was 5.0, 3.0, and 4.0 for nutrient solution, AMD, and diluted AMD, respectively. Seedlings did not establish in a hydroponics system supplemented with undiluted AMD, perhaps because it was too acidic (pH 3.0). So, we opted for the acidity of pH 4.0, which gave highly significant results (growth inhibition) compared with the nutrient control solution. In inductively coupled plasma - optical emission spectrometry (ICP–OES), as compared with nutrient solution, diluted AMD did not significantly alter Ca, Cr, Cu, K, Mg, Mn, Na, P, S, and Zn content. However, we found a subtle difference for Al and Fe that also could affect plant growth.

**Figure 1:**
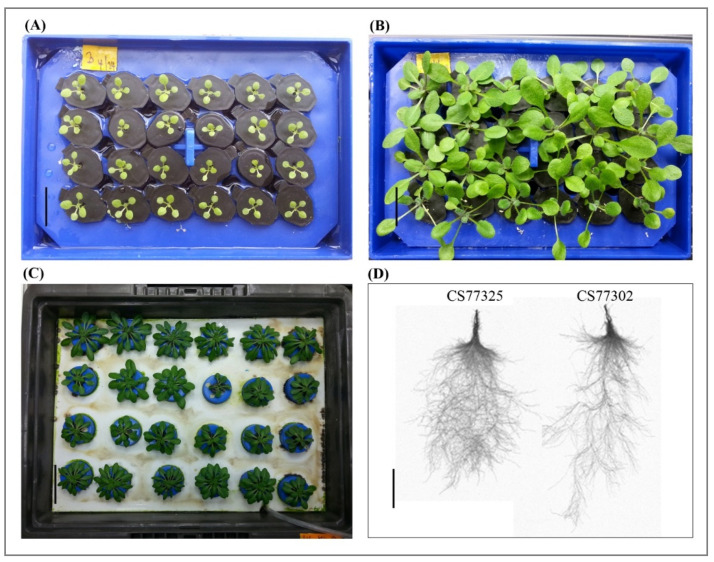
Hydroponics growth methodology adopted for phenotyping. Flow chart outlining the key steps in the process of growing seedlings till phenotyping. Seedlings were grown in small floating racks for (**A**) 10 days and (**B**) 21 days before moved into big trays for (**C**) another three weeks. Images of (**D**) 42-day-old seedlings were documented using WinRHIZO software. Scale: (**A**,**B**) 1 cm; (**C**) 3 cm; and (**D**) 4 cm.

The growth pattern among all ecotypes was uniform until we incrementally added diluted AMD to the experimental unit. Because we changed the medium completely with AMD, the seedlings varied in growth (Figure 1). These differences were observed more significantly after moving the 21-day-old seedlings into large well-aerated growth trays for another 21 days until we documented root traits with the WinRHIZO automatic root measurement system. We collected the data for five key traits, including primary root length, cumulative root length, root surface area, root volume, and root diameter.

### 2.2. Natural Variation in Root Growth Response to Acidity

**Figure 2:**
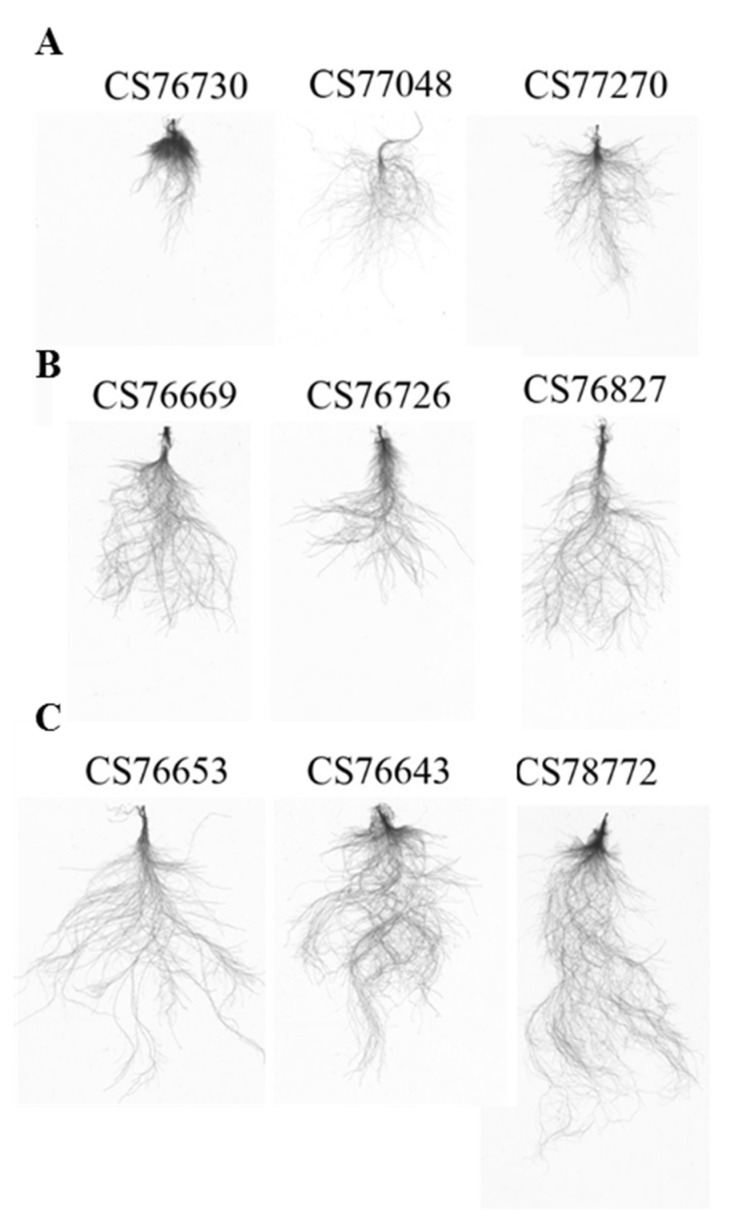
Natural variation of root growth pattern observed in *Arabidopsis* ecotypes. The WinRHIZO root scan images of randomly selected ecotypes with high phenotypic variation are presented. Representative images of (**A**) acid mine drainage (AMD)-sensitive, (**B**) moderately AMD-sensitive, and (**C**) AMD-tolerant *Arabidopsis* accessions are shown.

In response to AMD, root growth significantly varied among the 180 *Arabidopsis* ecotypes used in this study. From the phenotypic variation in root growth under AMD treatment, we classified the accessions as sensitive (CS76730, CS77048, CS77270, etc.), moderately sensitive (CS76669, CS76726, CS76827, CS77313, CS77321, CS77345, etc.) and tolerant (CS76653, CS76643, CS76810, CS77295, CS78772, CS78818, CS78832, etc.) (Figure 2). The performance of cumulative root length (length of all roots), root surface area, average diameter, root volume, and primary root length (from collar region to end of primary root) for the accessions ranged from 153 cm to 1533 cm, 8.1 cm^2^ to 133.8 cm^2^, 0.16 mm to 0.46 mm, 0.03 cm^3^ to 0.93 cm^3^, and 7.93 cm to 36.0 cm, respectively (Table S1). Variance explained by various treatments is in ANOVA (Table S2).

The median cumulative root length of plants grown in control was about 500 cm, and it was measured about 540 cm when the accessions were exposed to AMD treatment (Figure S1). The mean root length was 1000 cm and 1050 cm with control and AMD treatment for the top 20 performing accessions and was ~150 cm and 250 cm for the bottom 20 performers (Figure S1). The total, top 20, and bottom 20 performers were higher with AMD than a control treatment for cumulative and primary root length. For the whole population, measurements under control and AMD treatment were close for root surface area (~45 cm^2^) and root volume (~65 cm^3^). Root volume with AMD treatment was half that with control treatment for the bottom 20 performers. Overall, we found a considerable variation for all traits, with a few outliers.

### 2.3. GWAS by Using easyGWAS

Mixed linear models were selected based on genomic control (λ). Quantile–quantile (Q–Q) plots for various root traits are in Figure S2. Overall, Q–Q plots pertaining to AMD treatment fit best as compared with control treatment. Efficient mixed-model association expedited (EMMAX) [39] was used for detecting single nucleotide polymorphism (SNP) associations by using a principal component analysis-based population structure as a covariant to reduce spurious associations.

A total of 5651 and 5416 SNPs were significantly associated with all traits under control and AMD experiments, respectively; 771 SNPs were shared between the AMD and control conditions for all traits. Overall, 1032, 714, 1475, 1000, and 1430 SNPs were significant for cumulative root length, root surface area, root volume, average root diameter, and primary root length, respectively, under control conditions (Figure S3) and 1079, 1055, 1079, 1104, and 1099 SNPs under AMD conditions. We also found common SNP markers shared between the root traits under control and AMD conditions (Figure S3).

### 2.4. Candidate Genes Associated with Adaptation to AMD

GWAS revealed a set of potential genes operating simultaneously in a coordinated manner toward acidity stress. Details of SNP position, minor allele frequency, and strength of association for the top 20 SNPs/genes for each root trait are in Table S3. Our study focused on the top 20 SNPs (Table S4) for each of the five root traits based on the Bonferroni correction. For primary root length, we identified miR399b, NRAMP metal iron transporter, TIR-NBS-LRR disease resistance protein, vacuolar sorting protein 28 (VPS28), auxin-induced in root cultures protein 12 (AIR12), cation/H^+^ exchanger 13 (CHX13), and endomembrane trafficking Sec23/24. For cumulative root length, *miR399b*, *miR169n*, *DEAD helicase RAD3*, *CC-NBS-LRR* class, and *irregular xylem 11* (*IRX11*) genes were significantly associated. For the root surface area, SNARE protein, magnesium transporter CorA, heavy metal transport/detoxification family, ATP binding cassette A21 were identified. *C3HC4 zinc finger*, *miR399b*, and *DEAD helicase RAD3* genes showed a strong association with cumulative root length trait. Common factors were linked to root volume and root diameter—*miR399b*, *DEAD helicase RAD3*, *TIR-NBS-LRR*, and *CER26*. Four SNPs with a high *p*-value on chromosome 5 are associated with the auxin-responsive GH3 family, negatively regulating lateral root growth and shoot formation. Therefore, these stress-related genes are strongly associated with tolerance to AMD toxicity. The gene-specific minor and major alleles associated with total root variation in the hydroponic experiment indicated that these genes might play an important role in the plant tolerant response to unfavorable acidic growth regimes.

We used protein analysis through evolutionary relationships (PANTHER), a high-throughput classification system, to functionally classify the genes associated with GWAS. In this analysis, a binomial test [40] was used to identify over- or underrepresented terms in the sample gene set compared to the reference genome set (https://www.arabidopsis.org/). The gene ontology analysis was performed to understand the molecular function, biological process, and cellular component (Figure S4). Under molecular function, nearly 47% of genes belonged to catalytic activity, followed by a binding activity and transporter activity (Figure S4A). About 43% of transporters were located in the membrane region. The three major subcategories in the biological process were metabolic processes, cellular processes, and biological regulation (Figure S4B). Under cellular process, cell part, organelle, and macromolecular complex were the primary categories (Figure S4C). In total, 288 known proteins were classified as hydrolase, signaling, transferase, transport, and transcription factors (Figure S4D). These results suggested that these genes are vital for acidity tolerance.

### 2.5. Subcellular Location and Protein Interaction Network during Acidity Tolerance

Protein–protein interactions and protein–nucleic acid interactions are crucial for an organism to survive adverse stress conditions. To study interactions among close partners in *Arabidopsis* under AMD stress, we used the Genemania gene network integration tool (http://bar.utoronto.ca) [41] to build networks of the top 20 genes for each trait. Some genes were involved in transcription, membrane transporting, endomembrane trafficking, and metabolic activities. Interacting proteins should, in general, reside in the same subcellular location, although some will interact across adjacent subcellular locations, and some will migrate between compartments and could have interaction partners in both locations. The key regulators located in the vacuole or plasma membrane membranes are transporters and may be involved in transporting ions and metals.

The network analysis using the top 20 genes identified in GWAS for each trait showed strong co-expression and interactions with several other genes/proteins (Figure S5). The interaction and co-expression map for primary root length showed that these genes co-express together. For instance, the *AT4G18790* (*NRAMP5,* cation transmembrane transport gene), *At4g01380*, *At2g37700*, *At2g25430*, etc., which are among the top 20 genes show co-expression (Figure S5A). Also, four other *NARMP* genes—*NRAMP2, NRAMP3*, *NRAMP4*, and *NRAMP6*—are co-expressed with the top 20 genes associated with primary root length trait (Figure S5A). This result showed that these top 20 candidate genes might have an active role in cellular homeostasis to tolerate AMD-toxicity.

The top 20 genes associated with cumulative root length trait also showed co-expression patterns and several other genes (Figure S5B). The *At5g08620* (*RH25, DEA(D/H)-box RNA helicase* family protein gene) show co-expression with *At5g43740*, one of the top 20 genes. *RH25* also co-express with *ROOT UV-B SENSITIVE 4* and *At1g73180*. The *BR enhanced expression 3* (*BEE3*) showed co-expression with *At5g52890*, an *At hook motif-containing* protein gene, and *Serine carboxypeptidase-like 3* (Figure S5B). The genes identified for root surface area trait predicted for their functional interactions in the network analysis (Figure S5C). In this group, only two genes showed co-expression—*At1g12450* (*SNARE associated Golgi* protein gene) and *At1g68070* (*C3HC4 type (RING finger)* family protein gene) (Figure S5C). The top 20 genes associated with the root volume trait showed a co-expression pattern. *At1g13210* (*autoinhibited Ca^2+^/ATPase II*), *At4g13840* (*eceriferum 26; CER26*), *At2g14080* (*disease resistance protein* gene), *At4g39830* (*cupredoxin superfamily protein* gene), *At4g39120* (*myo-inositol monophosphatase like 2*), and *At4g39800* (*myo-inositol-1-phosphate synthase 1*) co-expressed together (Figure S5D). Interestingly, the co-expressed genes are involved in the phosphatase activity, sulfur metabolic process, and inositol phosphate phosphatase activity, implicating that they might have a critical role in AMD-stress tolerance.

For root diameter, we found that At5g13400, a transmembrane transporter gene, co-expressed with At5g08640 (flavonol synthase 1), and At4g13840 (eceriferum 26; CER26). Also, At3g52820 (purple acid phosphatase 22) and At4g13800 (magnesium transporter) show co-expression (Figure S5E). Interestingly, a set of four auxin-responsive GH3 family protein genes (At5g13350, At5g13360, At5g13370, and At5g13380) are among the top 20 candidate genes for root diameter. These genes co-expressed together, along with several other auxin-responsive genes (Figure S5E). Overall, the co-expressed genes are involved in auxin homeostasis, response to auxin, and ligase activity (Figure S5E), indicating that these genes are vital for plant survival under proton and AMD toxicity by modulating root architecture. These network analyses provide an avenue to find partners located in different subcellular compartments interacting for acidity tolerance.

### 2.6. miR399b and DNA helicase RAD3 Knockout Mutants Show Tolerance to AMD Toxicity

**Figure 3:**
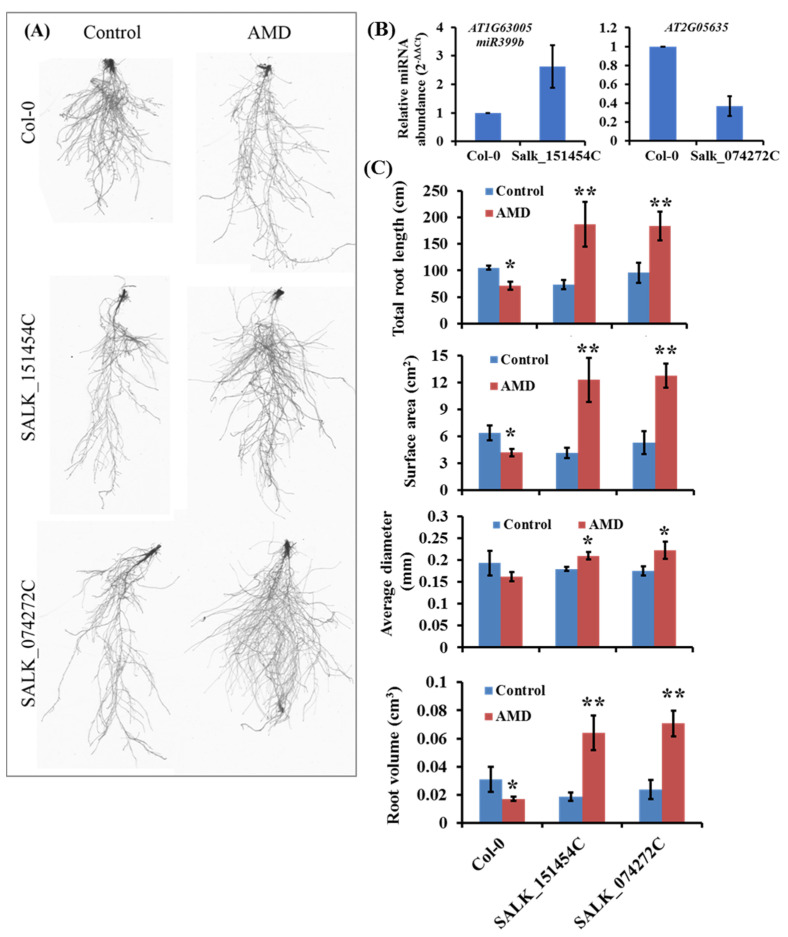
Phenotypic variation of T-DNA mutants of candidate genes in low pH stress condition. (**A**) Representative images of the phenotypic variation root length in wild type Col-0, Salk_151454C, and Salk_074272C T-DNA mutant seedlings in response to the control and AMD conditions. (**B**) Expression of *miR399b* and *DNA helicase RAD3* genes in the corresponding T-DNA insertion lines. (**C**) Quantitative analysis of phenotypic variation of root traits in Salk_151454C and Salk_074272C T-DNA mutant seedlings compared with Col-0 in response to the AMD toxicity. The single and double asterisks indicate the significance at *p* < 0.05 and *p* < 0.01, respectively, compared to the control.

The T-DNA insertion mutant lines for *miR399b* (*At1g63005*; Salk_151454C) and *DNA helicase RAD3* (*At2g05635*; Salk_074272C) were used to validate the role of these genes in response to AMD toxicity. Both T-DNA mutants showed increased root growth when grown on AMD versus control medium (Figure 3A). *miR399b* expression was induced in the T-DNA insertion line Salk_151454C (Figure 3B). We located the T-DNA insertion in the *At1g63005* promoter region (Figure S6), which could enhance the *miR399b* expression. However, the *miR399b* target genes under AMD toxicity stress remain to be uncovered. The transcript abundance of *DNA helicase RAD3* was significantly decreased in the Salk_074272C line, suggesting that T-DNA insertion has knocked out the *DNA helicase RAD3* (Figure 3B). Furthermore, these mutants showed a significantly enhanced root growth response to AMD in cumulative root length, surface area, and root diameter. The T-DNA mutant seedlings of both *miR399b* and *DNA helicase RAD3* showed significant tolerance to AMD toxicity. The quantitative analysis of root traits in the *miR399b* and *DNA helicase RAD3* mutant seedlings showed 3–4 times enhanced growth in response to AMD stress compared to Col-0 (Figure 3C). These results suggest that the *miR399b* and *DNA helicase RAD3* might have an important role in proton and AMD toxicity tolerance.

## 3. Discussion

This study used GWAS to uncover genomic loci in *Arabidopsis* associated with tolerance to AMD toxicity, which is a global problem in agricultural production. We characterized five major root phenotypes—cumulative root length, average root diameter, root surface area, root volume, and primary root length—in 180 *Arabidopsis* accessions in response to AMD-supplemented growth media. GWAS revealed genes associated with tolerance to AMD conditions. Most of these genes were transcription factors, anion/cation transporters, metal transporters, and unknown proteins. Two T-DNA insertion mutants showed enhanced acidity tolerance, especially *At1g63005* (*miR399b*) and *At2g05635* (*DEAD helicase RAD3*). Our GWAS and reverse genetic approach results suggested genes with tolerance to coal AMD.

Acidity stress synonymous with H^+^ rhizotoxicity or low-pH stress has long been a focus of research. Acid soil, defined as soil with pH < 5.5, is a global problem to agricultural production, and it hampers maize production up to 69% [42]. Acid soil toxicity is caused by a combination of heavy metal (Fe, Cu, Mn, Zn, and Al) toxicity, lack of essential nutrients (P, Mg, Ca, K, and Na), and acidity per se [43]. Low pH increases the solubility of metals such as Al, Fe, Mn, Zn, and Cu and makes large quantities unnecessarily available to plants as toxic nutrients [44]. When soil pH drops too low, Al becomes easily soluble and causes deleterious effects to crops [45]. Acidity and Al toxicity are prevalent and related toxicities because Al is only soluble under acidic environments. Low pH acidic soils can lead to Al, Mn, and Fe toxicities. Many research findings emphasized that pH toxicity is an important limiting factor in acidic environments, especially in very acidic soils. Practically, separating the effects of acidity per se from the direct effects of increased concentrations of toxic cations such as Al and Mn in an acidic environment is challenging.

The formation of a metal-rich acid solution known as acid mine drainage (AMD) is a major environmental problem associated with mining operations. With AMD exposure, the quality of adjacent surface water degrades greatly and eventually becomes unsuitable for sustaining biodiversity. The physicochemical properties of AMD from different types of mining operations varies highly, depending on the ore and metals. For example, in multi-metallic mining activity in China, the local soils were heavily polluted with Cd, Cu, and As, so the soil was unsuitable for crop cultivation [46]. ICP–OES findings and physicochemical properties revealed that AMD from coal mining had a significantly low pH and moderate Al and Fe (Table 1). Because low pH and Al, Fe, and Mn contents are tightly interrelated in AMD, we used AMD as one holistic experimental entity rather than conducting experiments for each factor individually.

Plants are sessile and cannot escape acidity stress; however, most plants can tolerate moderately acidic environments. The activity of H^+^-ATPase in the root cell plasma membrane becomes less effective to extrude protons into the apoplast under acidic conditions. However, both V-H^+^-ATPase and V-PPase relocate excessive H^+^ from the cytoplasm to vacuole [47] to maintain cytoplasmic pH (7.0–7.5) [48]. The efflux of protons regulates proton concentrations in the cytoplasm into the vacuole or the apoplastic space via proton pumps [49,50,51]. The phenomenon will be the same—accumulation or exclusion—with higher concentrations of metals. Mechanisms of plant tolerance to Al stress include exclusion of Al from the root tips and absorbance but tolerance of Al in root cells.

Naturally, some plant species have developed mechanisms to thwart acidity conditions, but others do not, even when pH reaches as low as 2.0. Some plant species, including *Acacia auriculiformis*, survive and nodulate at pH ≥ 2.0, and *Acacia confusa* and *Melaleuca armillaris* can survive at pH ≥ 2.5 [52]. Maize genotypes show genetic variability for tolerance to acidity, which can be exploited in developing high-yielding acid-tolerant maize genotypes [42]. In support of this concept, about 30% of 30 upland rice genotypes showed tolerance to soil acidity [53]. In these plants, nitrate (NTR1), ALMT1, and other transporters for heavy metals were upregulated to survive H^+^ rhizotoxicity. Continuous exposure to highly acidic growth conditions can significantly affect root cell structure and function [54].

In the present study, ~47% of candidate genes identified in GWAS were involved in catalytic functions. In total, 43 of 737 candidate genes were transporter protein genes, which implicates a membrane transporter role in cellular homeostasis. These genes include the heavy metal ATPases (HMAs), NRAMPs, the cation diffusion facilitator (CDF) family, the ZIP family, and cation antiporter proteins [55,56,57]. Furthermore, metal cation movement across membranes is predicted to be driven by the proton motive force generated by ATP-powered H^+^ pumps. Also, ABC superfamily proteins are primary pumps driven by ATP hydrolysis and transport a wide range of substrates, including ions, sugars, lipids, peptides, pigments, xenobiotics, and antibiotics. The *Arabidopsis* interactions viewer network analyses supported many interactions among partners from the nucleus, membrane transporters, vacuole, and endomembrane trafficking system.

Transcription factors are critical regulators during stress. The STOP1 transcription factor regulates At*ALMT1* in *Arabidopsis* under low pH [58]. GWAS revealed STOP1 was associated with root surface-area, specifically under acidity. The *stop1* mutant seedlings failed to produce roots under an acidic AMD environment (data are not shown), indicating that STOP1 controls the transcript abundance of a set of genes involved in low pH Al tolerance. Al^3+^-induced expression of the plasma membrane At*ALMT1* conferred Al stress tolerance [23]. Root cell wall modifications under Al toxicity play an important role in conferring Al stress tolerance [59]. *IRREGULAR XYLEM 11* (*KNAT7*) is a transcriptional repressor that negatively regulates transcription for xylem development and secondary cell wall biogenesis [60], and some family members—*KNAT1*, *KNAT3*, *KNAT4*, and *KNAT5*—play roles in regulating root development [61]. Therefore, the identification of a *KNAT* family gene in our analysis is no surprise. Modulation of the carbohydrate polymers in the root cell wall could be an adaptation strategy to excess Al in acidic growth conditions. Such cell wall modifications are shown to limit the binding of toxic Al^3+^ ions to the cell wall [62]. The Al retained in the root cell wall is transported to and sequestered into root cell vacuoles for storage [63]. Zinc finger protein tends to localize in the vacuole membrane and is an integral component of membrane and functions such as zinc ion binding. C3HC4-type RING finger proteins have been studied on a genomic scale in *Arabidopsis* and rice. *Arabidopsis* RING finger proteins with predicted or known biological functions include light signaling, secretory pathway, root development [64], and stress tolerance [65]. Identification of these genes in our analysis strengthens their functional roles in response to proton and Al stress under AMD toxicity.

MicroRNAs (miRNAs) are a class of ~22-nt short non-coding RNAs that play key roles in fundamental cellular processes, including how cells respond to environmental changes or stresses. *Arabidopsis* gene *At1g63005* encodes a phosphate starvation-responsive *miR399b* that targets PHO2, a ubiquitin-conjugating E2 protein that negatively affects shoot phosphate content. Phosphorus (P) is deficient in most acid soils because soluble inorganic P is fixed by Al and Fe [66]. The *At1g63005* mutant showed extensive root biomass under AMD treatment. However, *miR399b* target genes involved in conferring AMD toxicity tolerance and increased root biomass are still unknown. Recently, overexpression of *miR169* (*At3g26819*) conferred enhanced drought tolerance in tomato [67]. *miR169* also involved in regulates nodule formation in barrel clover [68], suggesting the miRNA functions in the growth-defense tradeoff. Therefore, identifying *miR399b* target genes associated with AMD stress tolerance and root growth is essential.

GWAS revealed several transporter or membrane proteins associated with AMD toxicity tolerance. Transporters play major roles in plant protection from many stresses. For instance, *AT4G18790* codes for natural resistance-associated macrophage protein (AtNRAMP5) is a divalent cation transporter for Fe^2+^ and Mn^2+^. A recent study found that streptococcal NRAMP homolog was crucial for the survival of *Streptococcus agalactiae* under acidic growth conditions [69]. These proteins likely localize on intracellular membranes such as the plastid envelope and the vacuolar membrane. In *Arabidopsis*, overexpression or disruption of *NRAMP* genes lead to changes in Fe or Cd sensitivity. The plant NRAMP family’s existence raises the question of the importance of dynamic metal compartmentalization in plant cells [70]. Natural variation underlies alterations in NRAMP aluminum transporter (NRAT1) expression and function that play a crucial role in rice aluminum tolerance [71]. As At*ALMT1* does in *Arabidopsis*, Gm*ALMT1* functions to adapt soybean under acid soils [72]. With high H^+^ rhizotoxicity, the root cell cytoplasm begins to acidify. Activated by both K^+^ transporters (AKT, HAK, and CHX13), H^+^-ATPase enables proton pump-driven extrusion from the cytoplasm into the apoplast. AtCHX13, a cation membrane proton antiporter, is located in the plasma membrane. AtCHX13 is involved in the endomembrane system to regulate pH [73]. PLASMODESMATA-LOCATED PROTEIN 7 (PDLP7) is an integral component of a membrane in plasmodesmata. PDLP7 has a role in adjusting plasmodesmata permeability for systemic acquired immunity in plants [74]. The function of PDLP7 could be related to H^+^ movement in and out of cells during the stress. Above all, we found a few heavy metal transporters or detoxification family members (*At3g04900* and *At4g16380*) associated with primary root length and root surface area.

Hormonal regulation of root modification occurs during acidity stress. Cell-wall acidification processes in line with the acid growth theory may also occur in roots. Auxin-induced loosening and growth of cell walls in roots are catalyzed by a group of enzymes known as expansins, which are also related to the plant responses to low pH stress [75]. During this reaction, cell-wall growth and extensibility are increased under low pH. Roots themselves can affect rhizosphere pH via influx or efflux of cations and anions, H^+^-ATPase activity, organic acid efflux, and root respiration. Another hormone regulator, BREVIS RADIX (*BRX*, *At1g31880*), is a positive regulator of auxin signaling required for the transient boost of polar auxin transport essential for root meristem growth [76]. An extracellular pH affects the auxin activity and cell proliferation rate in the root meristem in *Arabidopsis* mutants. The finding that proton pumps are hyperactive in *brx* roots could explain this phenomenon and is consistent with robust growth and increased fitness of *brx* mutants on overly acidic medium or soil. The novel *brx* allele in accessions recently collected from another acidic sampling site demonstrates the loss-of-function alleles in nature and supports that they are advantageous in acidic soil pH conditions [77]. GWAS also revealed a strong association with four auxin-responsive GH3 family proteins on chromosome 5, strongly associated with root diameter. DWARF IN LIGHT 1 (*DFL1*, *At5g54510*), an auxin-responsive GH3 gene homolog, negatively regulates shoot cell elongation and lateral root formation [78]. Also, auxin-induced in root cultures 12 (*AIR12*, *At3g07390*) is early expressed during auxin-induced lateral root formation in *Arabidopsis* [79]. GWAS also identified brassinosteroid (BR)-associated genes involved in AMD tolerance. BR enhanced expression 3 (BEE*3*) encoded by *At1g73830* is a DNA-binding transcriptional regulator activated by BR. *BEE* genes are negative regulators of physiological responses to salt stress and drought [80]. The hormonal regulation of low pH tolerance may be an important mechanism in plants. The role of these AMD-associated hormonal genes demands a further investigation for understanding their role in low pH stress adaptation.

Membrane organization and cell polarity is an important aspect of plant growth under stress. The endomembrane system is a series of compartments that pack, label, and ship proteins and molecules. Transport protein particle (TRAPP) may play a vital role in the targeting and/or fusion of endoplasmic reticulum-to-Golgi transport vesicles with their acceptor compartment. TRAPP is a large multimeric protein containing transporting activity. Vesicle trafficking controls the structures of intracellular compartments and communication between cells and the environment to transport proteins. The trans-Golgi network is a major hub of secretory and endocytic trafficking for transporting cellular cargo to the vacuoles through the endosomal sorting complexes required for transport (ESCRT) complex [81]. Vacuolar protein sorting 28 (VPS28) is part of the ESCRT I complex. The epsin N-terminal homology domain (ENTH, At2g25430) is a major player in clathrin-mediated endocytosis in root cells [82]. The endoplasmic reticulum is an essential organelle responsible for the synthesis, assembly, and sorting of secretory proteins. Protein transport between the endoplasmic reticulum and Golgi apparatus is mediated by COPI and COPII coat complexes. Sec23/24 (At4g14160) functions as part of COPII mediates protein transport [83]. Identifying these genes in our AMD GWAS analysis offers a new avenue to study the importance of cell-vesicle transport and ESCRT functions to maintain plant growth and development under acidic growth conditions.

The DNA repair *DEAD helicase RAD3* can regulate mitotic cell division, UV response, and oxidative stresses such as cold tolerance [84,85]. However, our analysis did not suggest that this helicase regulates profuse root growth in an acidic environment. A few LRR proteins were associated with root traits in our study: LRR family protein (*At4g13820*), disease resistance TIR-NBS-LRR (*At4g09430*), and CC-NBS-LRR (*At5g43740*). However, these genes have not been associated with acid tolerance.

As a member of acid phosphatases and located in the extracellular region, *At3g52820* (ATPAP22) is involved in dephosphorylation and metal ion binding in acidic medium to release an inorganic phosphate and plays a pivotal role in P metabolism [86]. Another member, BRG 1, has a molecular function of ubiquitin-protein transferase activity and metal ion binding and was linked to *Botrytis cinerea* disease resistance [87]. The involvement of BRG1 in AMD tolerance would be an interesting topic for research. Altogether, our results indicate that the candidate genes we selected based on GWAS have great potential to be tapped for rhizotoxicity research in plants.

## 4. Materials and Methods

### 4.1. Plant Materials and Collection of AMD and Analysis for Metals

We obtained 180 natural accessions of *A. thaliana* (Nordborg collections, from 1001 genome projects) from the Arabidopsis Biological Resource Center, Columbus, OH, USA (www.abrc.osu.edu). The experiments were carried out at the laboratory scale with AMD water samples collected from the waterhead of Cabin Creek near Dakota, WV, where AMD water seeps over the bank into the creek. The collected AMD water samples and AMD diluted with Hoagland’s nutrient solution were filtered through a 0.45-µM filter (MF-Millipore, Millipore Sigma, USA) before acidifying with concentrated HNO_3_ to pH < 2 and kept at 4 °C before analysis of total dissolved metals by using inductively coupled plasma-atomic emission spectroscopy (ICP–OES) (Perkin Elmer, Waltham, MA, USA).

### 4.2. Hydroponic Culture Growth Conditions

The seedlings were grown in a modified hydroponic system after a few modifications to the existing protocol [88]. Each ecotype was grown in three replications in uniform hydroponic growth conditions for efficient phenotyping. Germination medium was prepared in distilled water as described [88]. The lid of a black microcentrifuge tube with a 1.2-mm to 1.8-mm diameter hole was filled with ~450-µL of 0.7% agarose gel in the germination medium to have a dome shape. The lids with solidified agar were kept inverted in floating racks (FR24, MIDSCI, St. Louis, MO, USA), ensuring that the agar dome contacted the germination medium. Seeds were sown on agarose gel through the small hole and were stratified at 4 °C in a dark and humid condition for 48 h. The floating racks were then transferred to 22 °C under 16-h:8-h light: dark cycles. The medium was incrementally changed from germination medium to Hoagland’s solution (pH 5.0; control) then to diluted AMD water (pH 4.0) in three days. The 21-day-old seedlings were transferred to a large, aerated hydroponics tank containing 10 L Hoagland’s medium for control and 10 L diluted AMD for the experiment. The 50-mL falcon tubes were modified by making a hole of 11 mm in diameter in the center of the cap to support the germination lid and cutting the bottom end to allow roots to grow freely in the medium. Finally, 21-day old seedlings were carefully transferred to Falcon tubes and left for another 21 days to grow further with the intermittent change of diluted AMD before measuring root traits.

### 4.3. Documentation of Phenotypic Traits and Statistical Analysis

The measurement of four phenotypic traits (cumulative root length, root volume, average root diameter, and root surface area) was obtained by using WinRHIZO pro-2012 b software (Regent Instruments Inc., Quebec, Canada; http://regentinstruments.com). The primary root length of individual plants was measured manually from the collar region to the end of primary roots. All quantitative data for five phenotypic traits were statistically analyzed by one-way ANOVA to partition the variance in the population.

### 4.4. GWAS

GWAS involved using a cloud-based platform called easyGWAS [89]. All phenotypic traits were recorded and assembled in a Plink format allowed by easyGWAS. The genotypic data for selected *Arabidopsis* accessions from 1001 genomes were available in easyGWAS. The efficient mixed-model association expedited (EMMAX) method [39] was used for GWAS, which takes population structure into account by using a genetic relatedness matrix.

### 4.5. Gene Ontology Enrichment and Coexpression Network Analysis

The protein analysis through evolutionary relationships (PANTHER) database was used to classify the GWAS-identified candidate genes (www.pantherdb.org). The genes were classified into three categories—molecular function, biological process, and cellular component. Co-expression network analysis of the GWAS candidate genes involved using the Genemania gene network integration tool (http://bar.utoronto.ca) [41].

### 4.6. Genotyping of T-DNA Mutants and Phenotypic Validation

Loci of strongly associated SNPs were identified, and T-DNA mutants for the specific loci/genes (Table S3) were requested from the *Arabidopsis* Biological Resource Center. The mutants were genotyped by using locus-specific [Salk_151454C Left Primer (LP) and Right Primer (RP), Salk_074272C LP and RP and T-DNA border primer (left border primer LBb1.3) sets (http://signal.salk.edu/) (Table S4). Homozygous T-DNA mutants were grown in a hydroponic system and scanned by using WinRHIZO to obtain phenotypic data. Data from three replications were used to draw significant comparisons to the control with the Student’s t test.

### 4.7. Complementary DNA (cDNA) Synthesis and RT-qPCR

cDNA was synthesized by first-strand cDNA synthesis with Superscript II RT (Invitrogen). A 20.0-µL reaction volume was set by adding 1.0 µg total RNA in 5.0 µL (concentration of 200 ng/µL), 1.0 µL Oligo(dT), 1.0 µL dNTP Mix (10 mM each), and 5.0 µL sterile distilled water for a total volume of 12.0 µL. The mixture was heated at 65 °C for 5 min and quickly chilled on ice. To the above contents was added 4.0 µL of 5X first-strand buffer, 2.0 µL 0.1 M DDT, and 1.0 µL RNase out. The ingredients were mixed gently by pipetting and incubated at 42 °C for 2 min. After incubation, 1.0 µL (200 units) Superscript II RT was added and mixed gently with the pipette and incubated at 42 °C for 90 min. After incubation, the tubes were heated at 70 °C for 15 min for reaction inactivation. Finally, 1.0 µL (2 units) RNase H was added and incubated at 37 °C for 20 min to remove extra RNA. The cDNA synthesized was used for RT-qPCR for gene expression profiles of selected candidate genes and endogenous control *Actin8*.

## 5. Conclusions

This study identified genetic loci responsible for acidity tolerance using a GWAS approach in the model plant *Arabidopsis*. Few accessions outperformed other ecotypes in response to AMD stress, so proton resistance in hydroponic conditions could be an important index in breeding programs to improve acid soil productivity, at least in acid-sensitive plant species [90]. GWAS results were validated by using T-DNA insertion mutants of selected genes. The T-DNA mutant seedlings of *miR399b* and *DEAD helicase RAD3* showed a strong association with most of the root traits under AMD-toxicity stress. Root growth phenotypes were enhanced in response to AMD stress, so *miR399b* and *DEAD helicase RAD3* are involved in the growth-defense tradeoff in plants under acidic stress conditions. Moreover, identifying several acidity-specific genes and pathways demands further investigation into their roles in enhancing acidity tolerance in plants. Therefore, this study provided a pivotal platform to use these genes in genetic engineering approaches and marker-assisted breeding. Our results can help in developing phytoremediation, which is the emerging passive AMD treatment technology for AMD-impacted soil or water. The future study demands the characterization of genes identified in this study to uncover their role in AMD toxicity tolerance. Additionally, we would like to study different targets of *miR399b* for acidity tolerance. To conclude, this study identified vital transporters, transcription factors, metabolic enzymes, hormone-signal regulating genes that could modulate root architecture to confer low pH stress tolerance in plants.

## Figures and Tables

**Table 1:** The results of inductively coupled plasma - optical emission spectrometry (ICP-OES) analysis of acid mine drainage and nutrient solution.

**Sample**	**pH**	**EC ^#^**	**Al**	**As ^@^**	**Ba ^@^**	**Ca**	**Cd ^@^**	**Co ^@^**	**Cr**	**Cu**	**Fe**
**Nutrient Solution**	5.00 ± 0.03	1601 ± 3.61	0.295 ± 0.091	<0.795	<0.020	47.8 ± 1.07	<0.051	<0.090	0.158 ± 0.003	0.068 ± 0.006	3.09 ± 0.16
**AMD**	3.03 ± 0.01 **	1459 ± 2.65 *	21.2 ± 0.453 **	0.032 *	0.025 ± 0.000 *	117 ± 4.6 **	<0.051	<0.090	0.160 ± 0.000	0.138 ± 0.010 **	53.0 ± 1.6 **
**Diluted AMD**	4.01 ± 0.01 **	1597 ± 2.31	0.333 ± 0.142 *	<0.795	<0.020	47.1 ± 2.36	<0.051	<0.090	0.160 ± 0.000	0.062 ± 0.003	3.19 ± 0.30 *
**Sample**	**K**	**Mg**	**Mn**	**Mo ^@^**	**Na**	**Ni ^@^**	**P**	**Pb ^@^**	**S**	**Se ^@^**	**Zn**
**Nutrient Solution**	242 ± 6.99	72.2 ± 0.32	0.82 ± 0.02	<0.210	42.5 ± 2.61	<0.23	19.8 ± 0.30	<0.630	82.7 ± 1.2	<1.13	1.69 ± 0.02
**AMD**	14.7 ± 0.72 **	83.1 ± 3.16 *	9.98 ± 0.16 **	<0.210	32.6 ± 2.37 **	<0.225	0.283 ± 0.006 **	<0.630	364 ± 17 **	<1.125	0.457 ± 0.003 **
**Diluted AMD**	240 ± 2.7	71.7 ± 1.22	0.81 ± 0.032	<0.210	43.3 ± 2.11	<0.225	20.2 ± 0.94	<0.630	82.9 ± 2.7	<1.125	1.67 ± 0.10

^#^—μS cm^−1^; all metals: mg/L; ^@^—below method quantification limit; EC—the solution electrical conductivity; —a measure used to assess the total salt concentration. The single and double asterisks indicate the significance at *p* < 0.05 and *p* < 0.01, respectively.

## Data Availability

Not applicable.

## Supplementary Materials

The following are available online at https://www.mdpi.com/2223-7747/10/2/191/s1, Figure S1: Natural variation was observed for different root traits. Effect sizes for total accessions, top 20, and bottom 20 accessions for cumulative root length, root surface area, root volume, root diameter, and primary root length in control (left panel) and acid mine drainage water (right panel). Yellow and blue indicate a higher and lower reduction in biomass associated with the Col-0 allele, respectively. Box plots represent the median value indicated by a thick red line, Figure S2: Q–Q plots of different root traits. The plots were drawn for expected vs. observed -log10 (p-values) for cumulative root length, root surface area, root volume, average root diameter, and primary root length both in controlled and AMD conditions, Figure S3: Common SNP markers between and among multiple root traits. Highly significant markers were taken for analysis to identify commonly associated SNPs among multiple traits across different traits for (A) control and (B) AMD, Figure S4: Gene ontology enrichment by PANTHER classification of the 737 genes representing significant SNPs analyzed for (A) molecular function, (B) biological process, (C) cellular components, and (D) further classification of the molecular functions of the genes, Figure S5A: Gene network interactions of top 20 candidates associated with primary root length, Figure S5B: Gene network interactions of top 20 candidates associated with cumulative root length, Figure S5C: Gene network interactions of top 20 candidates associated with root surface area, Figure S5D: Gene network interactions of top 20 candidates associated with average root volume trait, Figure S5E: Gene network interactions of top 20 candidates associated with average root diameter trait, Figure S6: Pictorial diagram showing T-DNA insertion positions on AT1G63005 and AT2G05635 genes, Table S1: Top five ecotypes showed the highest and lowest root phenotypic variation, Table S2: Analysis of variance (ANOVA) of multiple root traits, Table S3: Top 20 significant SNPs associated with different root traits, Table S4: Primers used for genotyping and RT-qPCR analyses in this study.
