# Supplementary material for: Genome-Wide Association Study of Natural Variation in Arabidopsis Exposed to Acid Mine Drainage Toxicity and Validation of Associated Genes with Reverse Genetics
Source: Plants (Basel). 2021 Jan 20;10(2):191. doi: 10.3390/plants10020191 (PMC7909446; doi:10.3390/plants10020191)
Supplement: Supplementary file 1 [file plants-10-00191-s001.pdf]

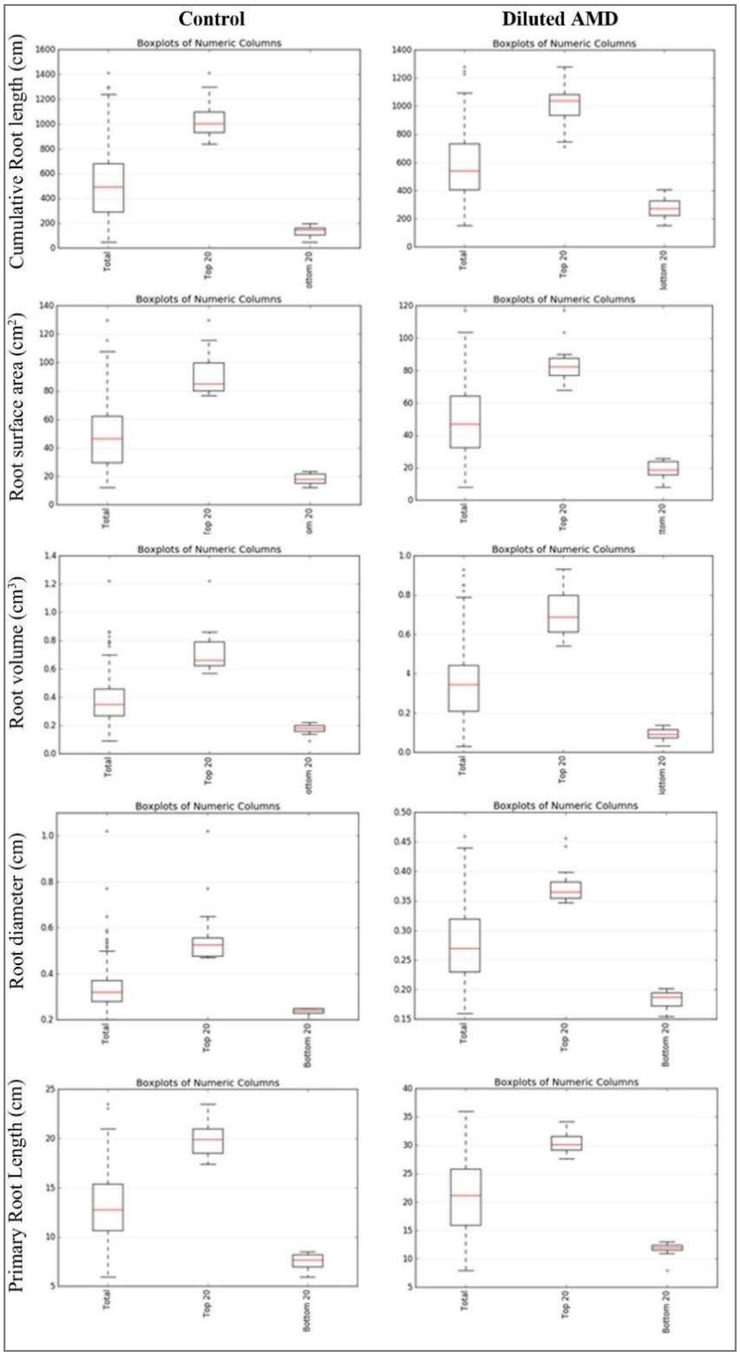


**Figure S1.** Natural variation was observed for different root traits. Effect sizes for total accessions, top 20 and bottom 20 accessions for cumulative root length, root surface area, root volume, root diameter, and primary root length in control (left panel) and acid mine drainage water (right panel). Yellow and blue indicate a higher and lower reduction in biomass associated with the Col-0 allele, respectively. Box plots represent the median value indicated by a thick red line.

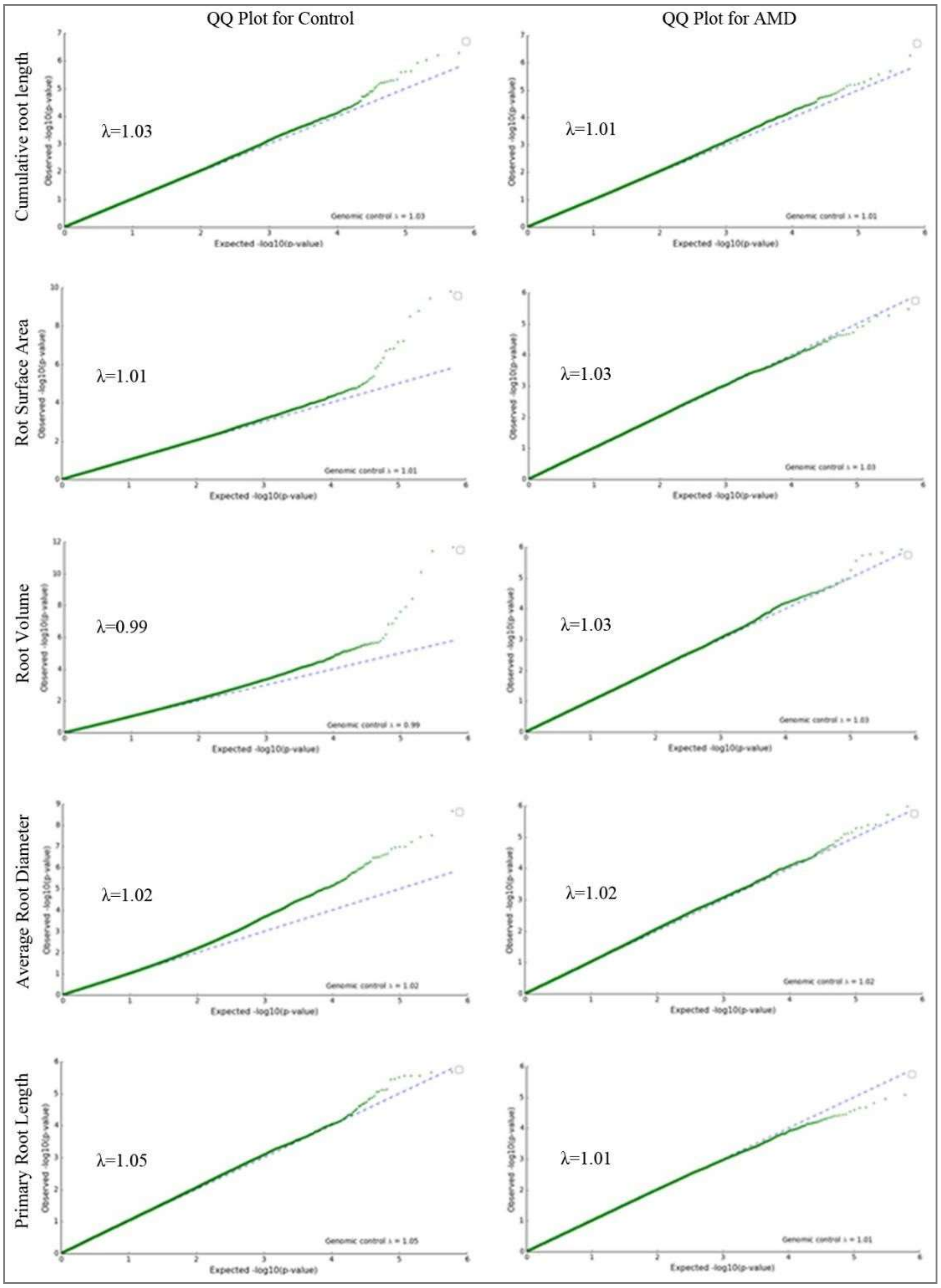


**Figure S2.** Q-Q (quantile-quantile) Plots of Different Root Traits. The plots were drawn for expected vs. observed -log10 (*p*-values) for cumulative root length, root surface area, root volume, average root diameter, and primary root length both in controlled and AMD conditions.

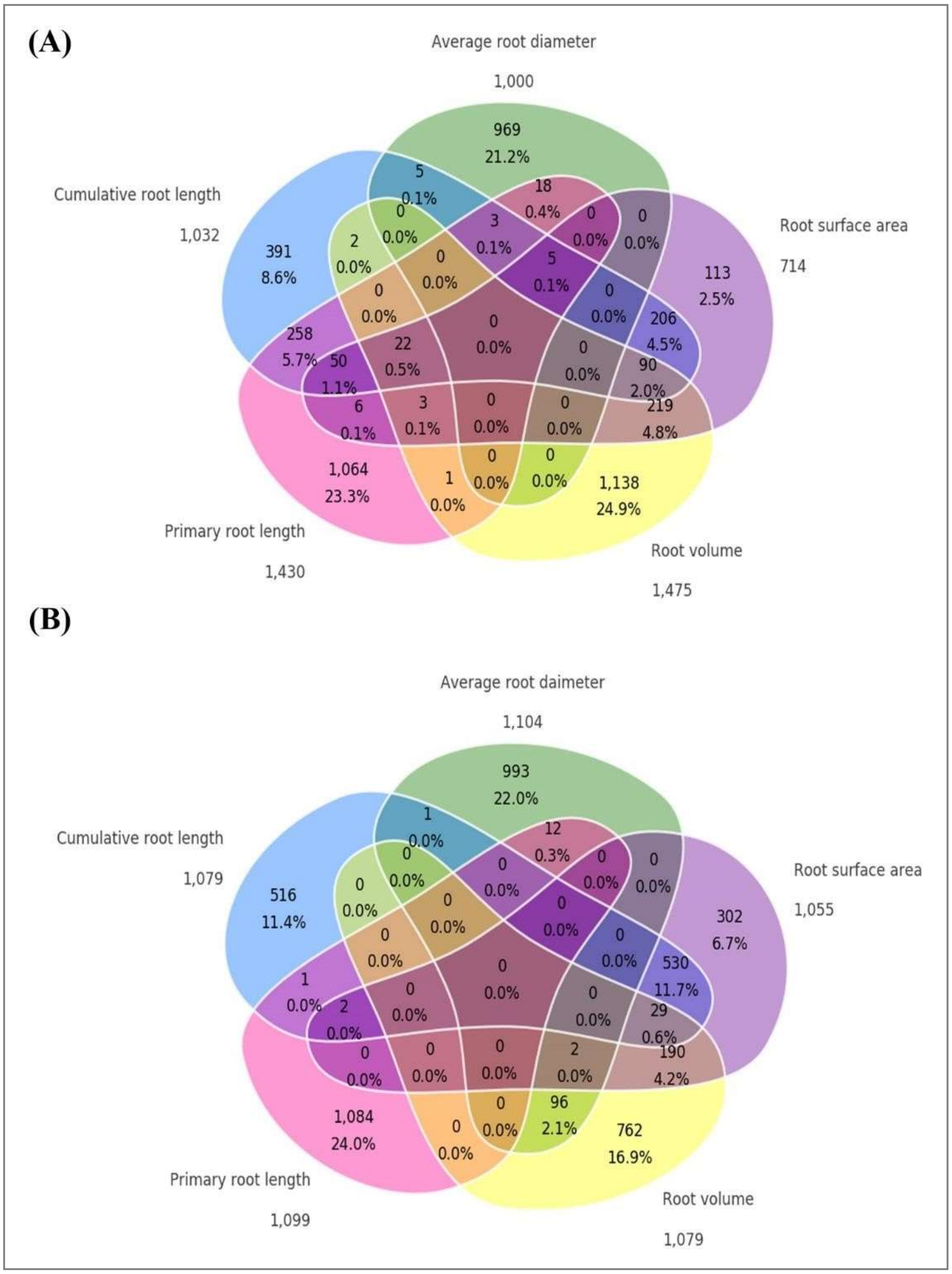


**Figure S3.** Common SNP markers between and among multiple root traits. Highly significant markers were taken for analysis to identify commonly associated SNPs among multiple traits across different traits for control (**A**) and AMD (**B**).

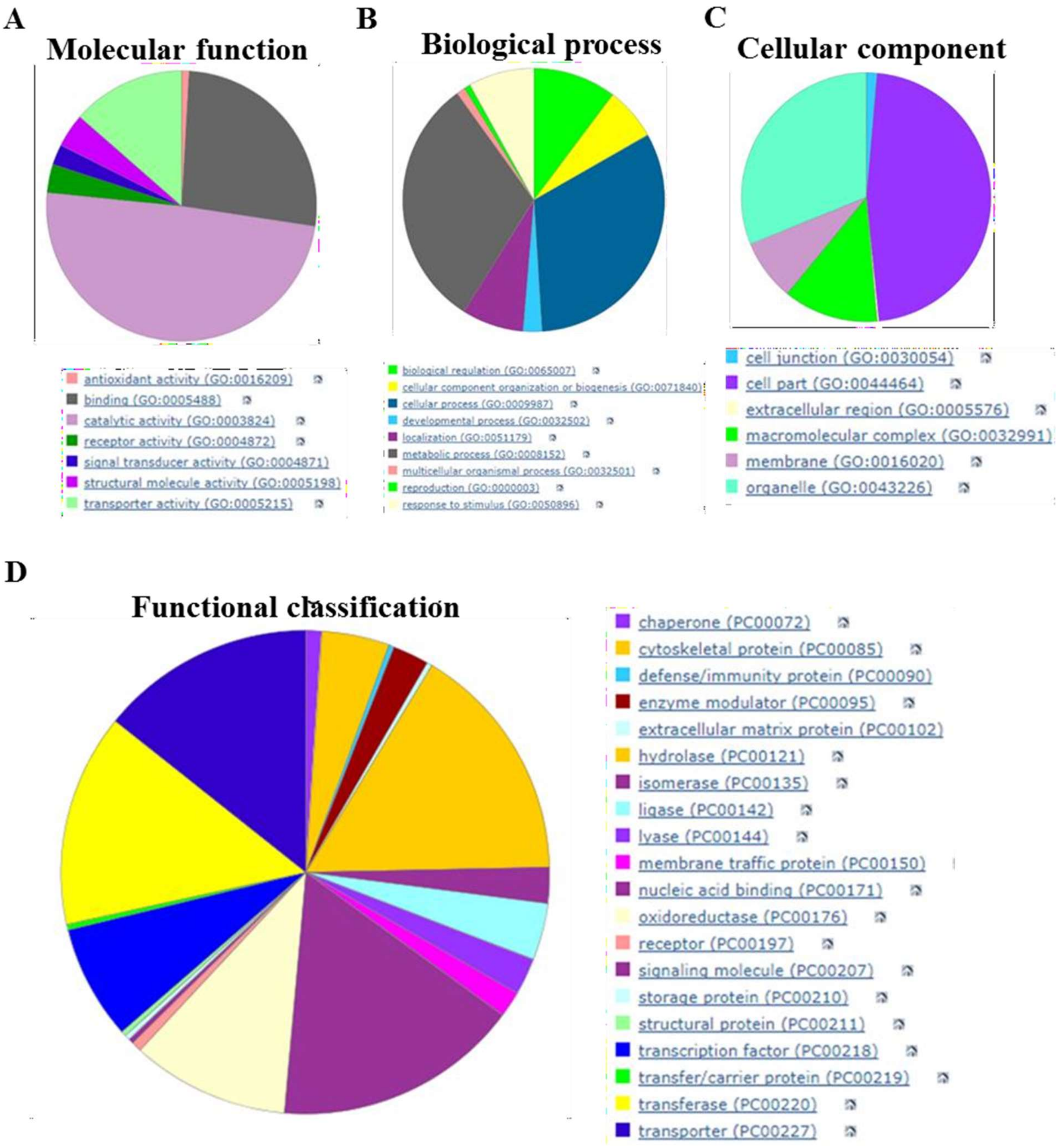


**Figure S4.** Gene ontology enrichment by Panther classification of the 737 genes representing significant SNPs analyzed for molecular function (**A**), biological process (**B**), cellular components (**C**). Further classification of the molecular functions of the genes (**D**).

**A**

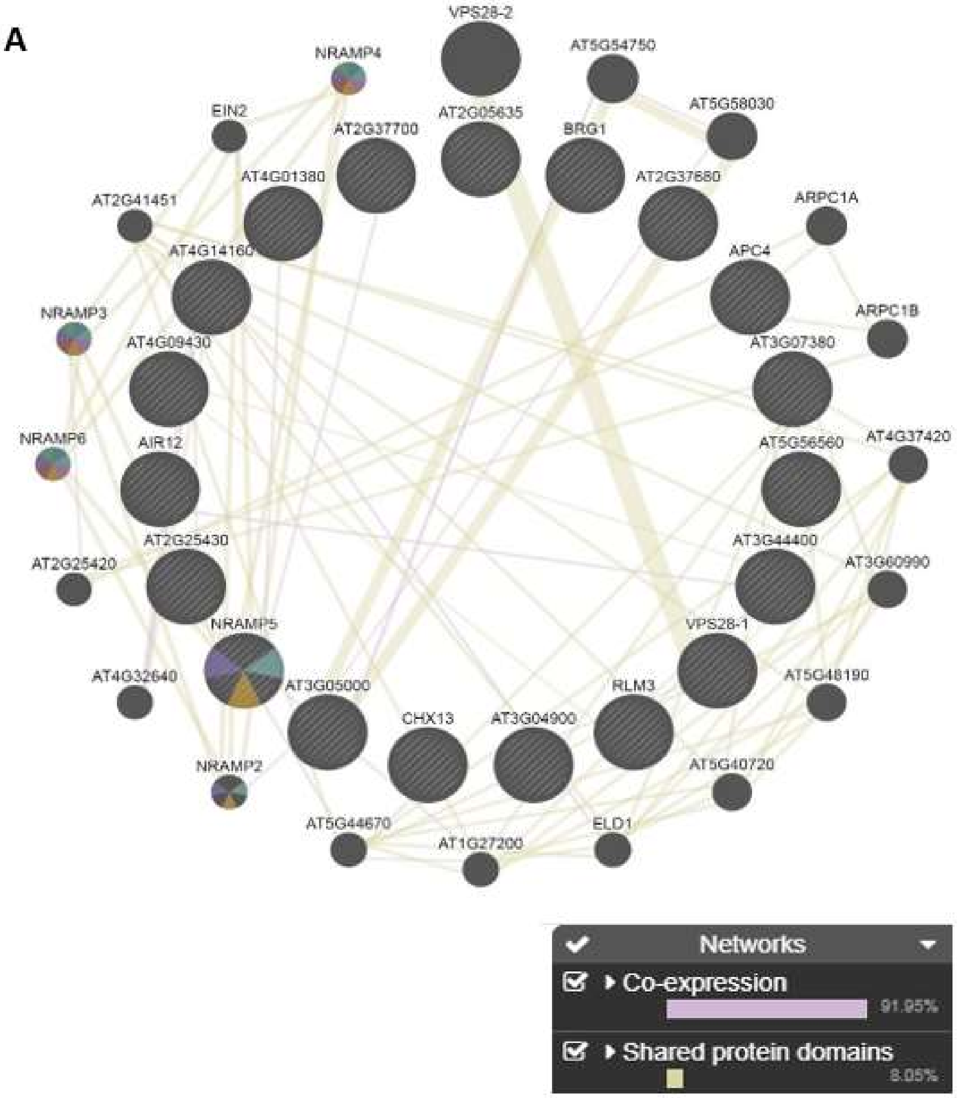


| Function | FDR | Coverage |
|---|---|---|
| inorganic anion transmembrane transporter activity | 3.04e-4 | 5 / 49 |
| cellular cation homeostasis | 5.03e-4 | 5 / 67 |
| cellular metal ion homeostasis | 5.03e-4 | 5 / 64 |
| cellular ion homeostasis | 7.14e-4 | 5 / 76 |
| cellular chemical homeostasis | 9.46e-4 | 5 / 84 |
| metal ion homeostasis | 1.18e-3 | 5 / 91 |
| cellular homeostasis | 1.25e-3 | 5 / 95 |
| cation homeostasis | 1.35e-3 | 5 / 99 |
| ion homeostasis | 2.42e-3 | 5 / 114 |
| chemical homeostasis | 8.42e-3 | 5 / 150 |
| metal ion transmembrane transporter activity | 9.27e-3 | 5 / 156 |
| metal ion transport | 1.05e-2 | 5 / 163 |
| homeostatic process | 1.26e-2 | 5 / 172 |
| divalent metal ion transport | 1.62e-2 | 3 / 27 |
| anion transmembrane transporter activity | 1.62e-2 | 5 / 184 |
| divalent inorganic cation transport | 1.90e-2 | 3 / 29 |
| inorganic cation transmembrane transporter activity | 1.96e-2 | 5 / 199 |
| transition metal ion transmembrane transporter activity | 2.74e-2 | 3 / 34 |
| cation transport | 3.03e-2 | 5 / 223 |
| transition metal ion transport | 7.48e-2 | 3 / 49 |

**Figure S5. A.** Gene network interactions of top 20 candidates associated with primary root length trait.

B

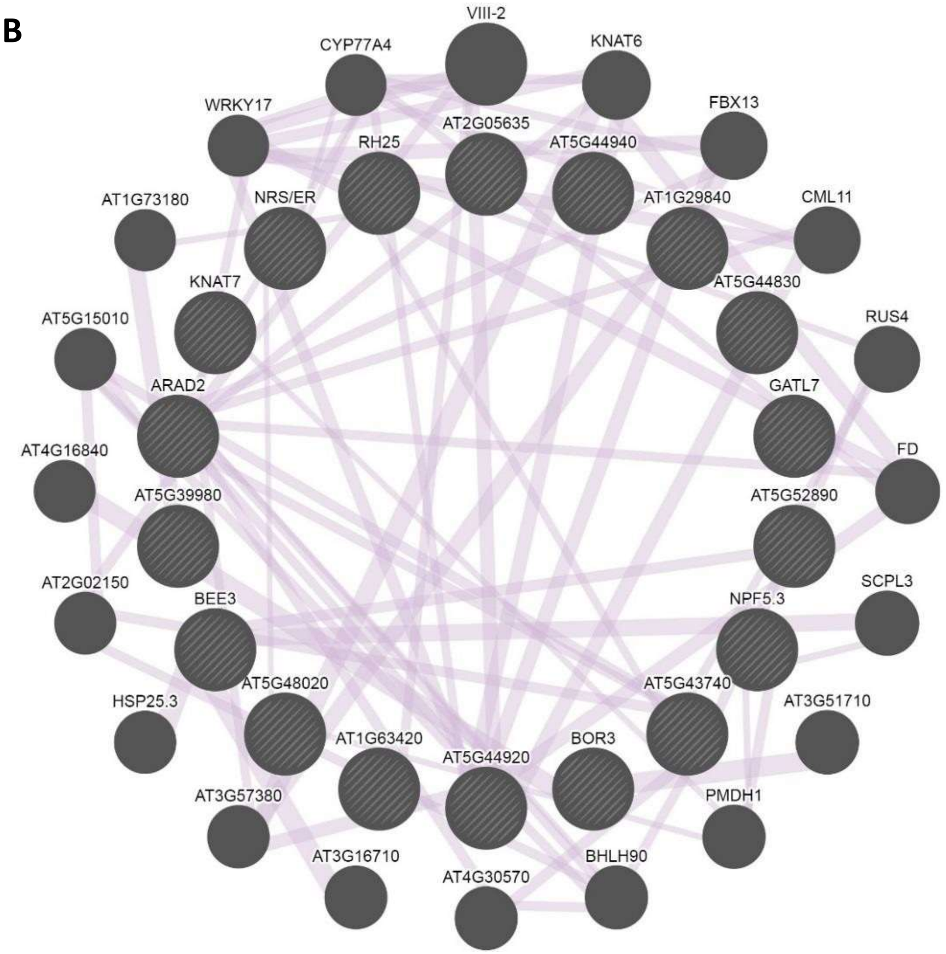


**Figure S5. B.** Gene network interactions of top 20 candidates associated with cumulative root length trait.

**C**

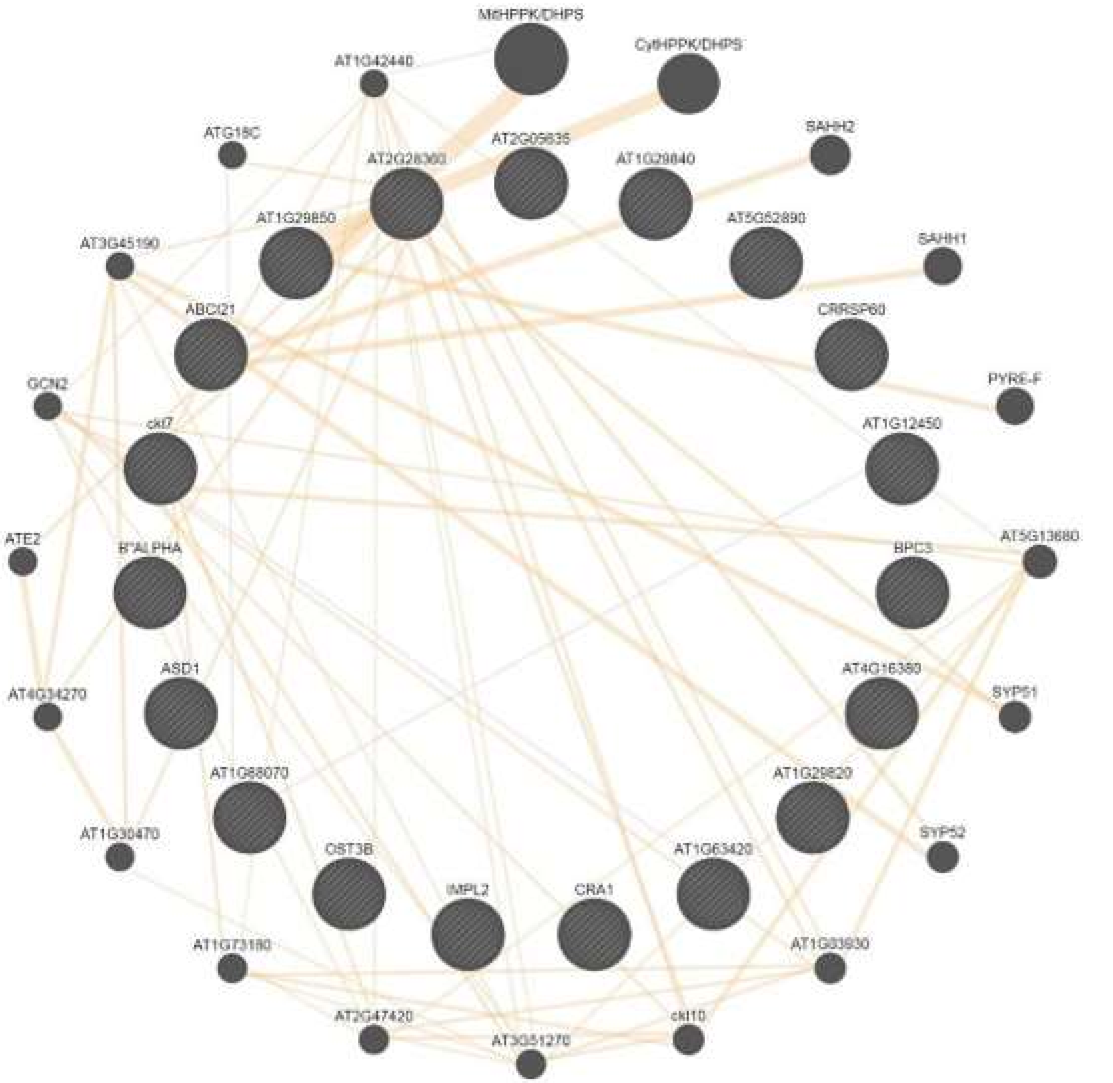


**Figure S5. C.** Gene network interactions of top 20 candidates associated with root surface area trait.

**D**

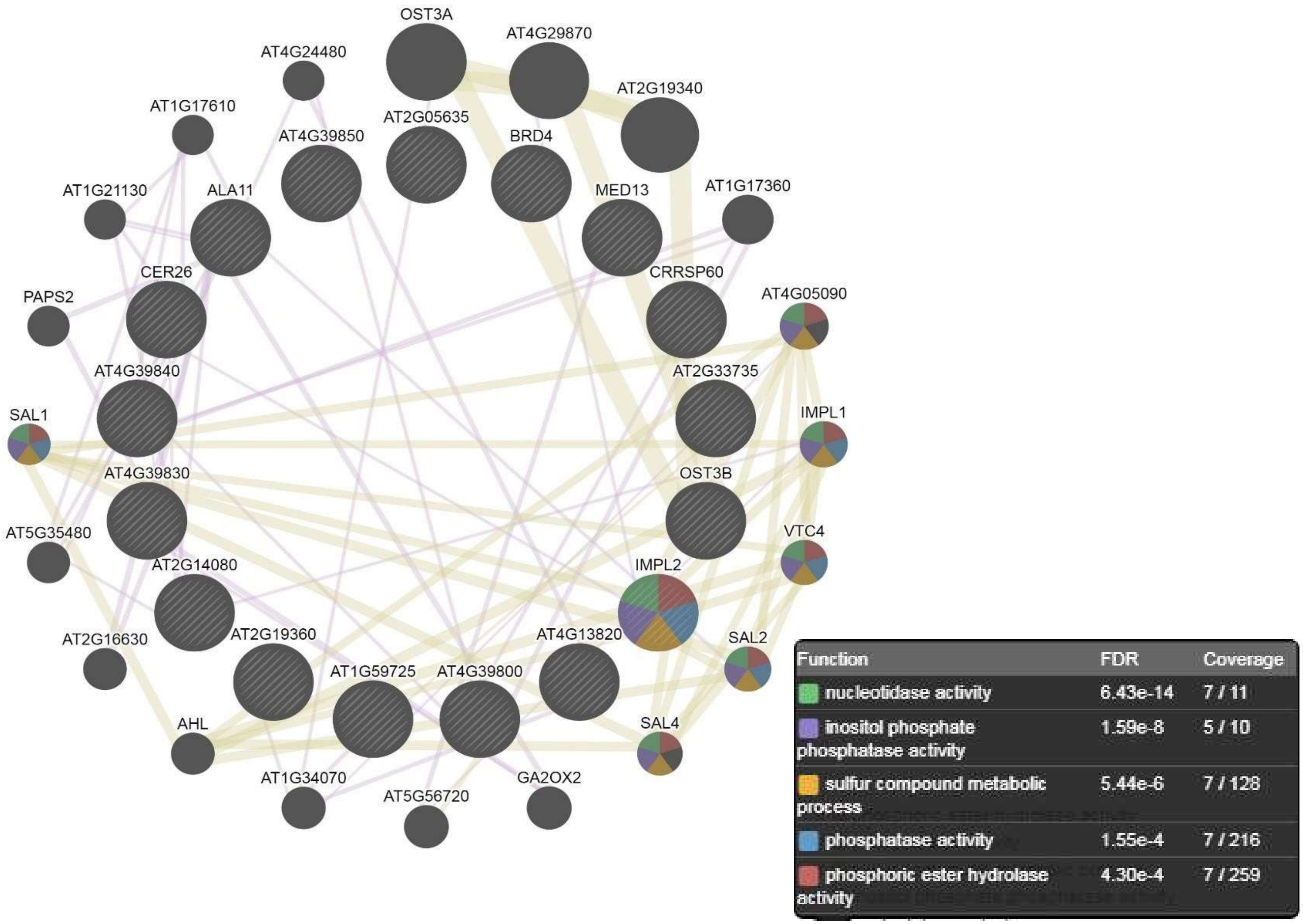


**Figure S5. D.** Gene network interactions of top 20 candidates associated with root volume trait.

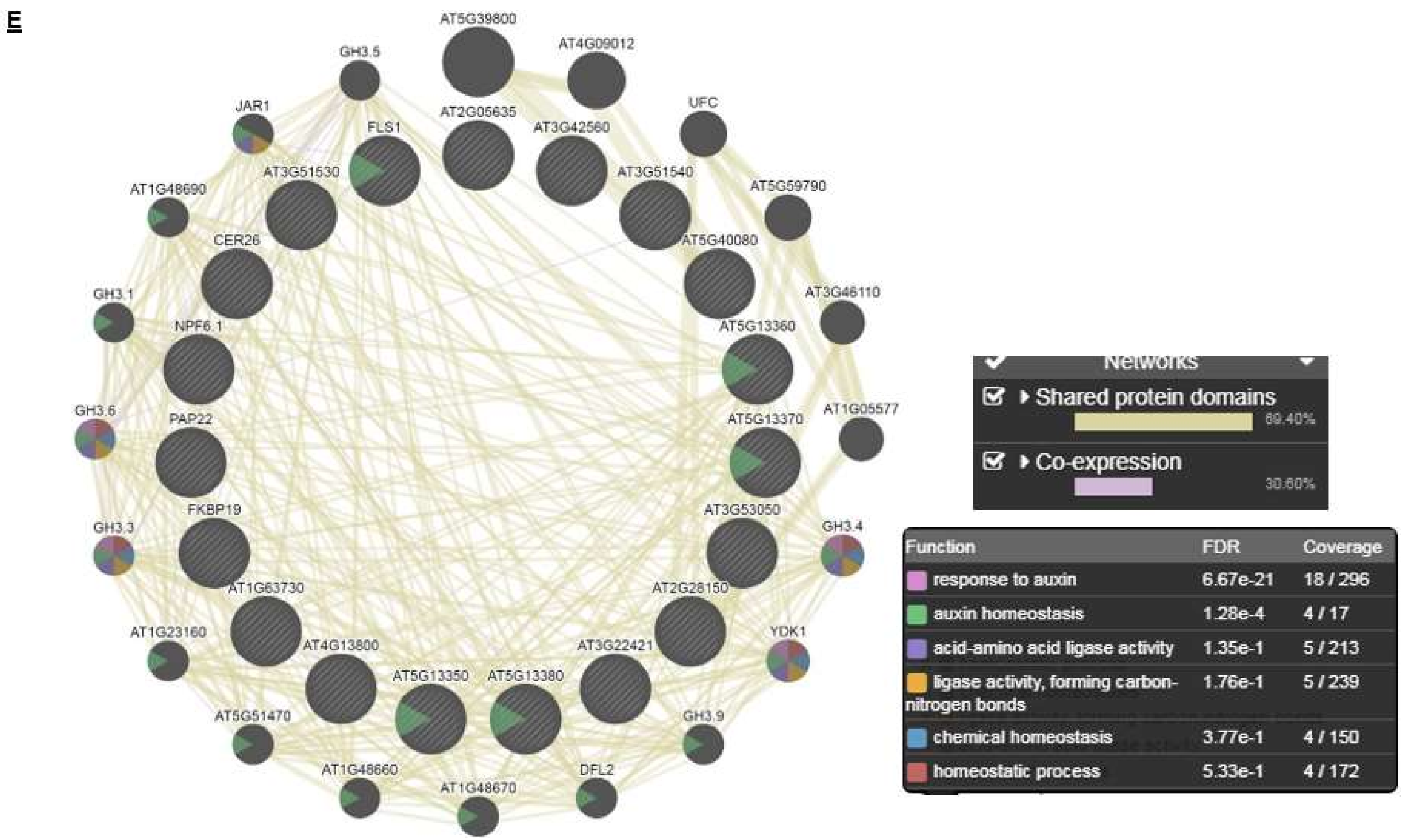


**Figure S5. E.** Gene network interactions of top 20 candidates associated with average root diameter trait.

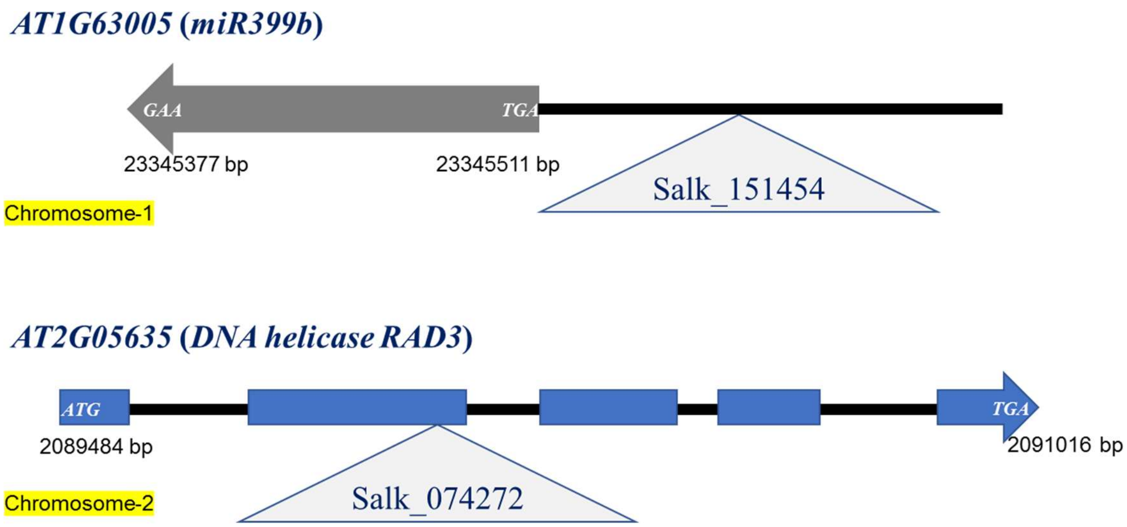


**Figure S6.** Pictorial diagram showing T-DNA insertion positions on *AT1G63005* and *AT2G05635* genes.

**Table S1.** Top five ecotypes showed highest and lowest root phenotypic variation.

| Ecotypes | Cumulati v e Root Length (cm) | Genotyp e | Surface area ($cm^2$) | Genotyp e | Ave. Diameter (mm) | Genotyp e | Root Volume ($cm^3$) | Genotyp e | Primary Root length (cm) |
|---|---|---|---|---|---|---|---|---|---|
| | | | | **Superior Performers** | | | | | |
| **CS77315** | 1533 | CS77315 | 133.8 | CS77299 | 0.46 | CS77315 | 0.93 | CS77009 | 36.00 |
| **CS76643** | 1281 | CS77320 | 117.0 | CS77136 | 0.46 | CS77320 | 0.93 | CS76861 | 34.17 |
| **CS78772** | 1250 | CS78818 | 103.5 | CS77137 | 0.44 | CS78818 | 0.90 | CS76932 | 32.50 |
| **CS78769** | 1227 | CS78830 | 99.2 | CS77270 | 0.40 | CS77145 | 0.85 | CS76863 | 32.43 |
| **CS77348** | 1096 | CS77146 | 90.1 | CS77060 | 0.39 | CS77146 | 0.85 | CS76986 | 32.23 |
| | | | | **Inferior Performers** | | | | | |
| **CS77359** | 153 | CS76908 | 14.6 | CS76806 | 0.17 | CS76834 | 0.07 | CS77268 | 11.47 |
| **CS77048** | 162 | CS76834 | 13.8 | CS76830 | 0.17 | CS76826 | 0.07 | CS77275 | 10.93 |
| **CS76827** | 182 | CS77359 | 10.6 | CS76826 | 0.16 | CS76908 | 0.06 | CS77304 | 10.17 |
| **CS77050** | 203 | CS77048 | 10.0 | CS76908 | 0.16 | CS77048 | 0.04 | CS77305 | 9.87 |
| **CS77289** | 206 | CS76827 | 8.1 | CS76827 | 0.16 | CS76827 | 0.03 | CS77048 | 7.93 |

**Table S2.** Analysis of variance (ANOVA) of multiple root traits.

| ANOVA | | | | | | |
|---|---|---|---|---|---|---|
| | | Sum of Squares | df | Mean Square | F | Sig. |
| Length | Between Groups | 30938771.438 | 164 | 188651.045 | 18.155 | 0.000 |
| | Within Groups | 3740785.944 | 360 | 10391.072 | | |
| | Total | 34679557.383 | 524 | | | |
| Surfacearea | Between Groups | 226174.790 | 164 | 1379.115 | 14.524 | 0.000 |
| | Within Groups | 34184.548 | 360 | 94.957 | | |
| | Total | 260359.338 | 524 | | | |
| Avgdia | Between Groups | 1.875 | 164 | 0.011 | 7.331 | 0.000 |
| | Within Groups | 0.562 | 360 | 0.002 | | |
| | Total | 2.437 | 524 | | | |
| RootVol | Between Groups | 16.818 | 164 | 0.103 | 10.266 | 0.000 |
| | Within Groups | 3.596 | 360 | 0.010 | | |
| | Total | 20.414 | 524 | | | |
| ShootFW | Between Groups | 464.626 | 164 | 2.833 | 11.423 | 0.000 |
| | Within Groups | 89.287 | 360 | 0.248 | | |
| | Total | 553.913 | 524 | | | |
| RootL | Between Groups | 19377.801 | 164 | 118.157 | 15.874 | 0.000 |
| | Within Groups | 2679.672 | 360 | 7.444 | | |
| | Total | 22057.474 | 524 | | | |

**Table S3.** Top 20 significant SNPs associated with different root traits.

| **Primary root length** | | | | | | | | |
|---|---|---|---|---|---|---|---|---|
| **Chr** | **Position** | ***p*-value** | **TestStat** | **Beta0** | **Beta1** | **MAF** | **Gene** | **Gene function** |
| Chr1 | 23345414 | 5.78e-06 | 10.972 | 23.014 | 3.821 | 0.221 | At1g63005 | Microrna399b |
| Chr4 | 8169552 | 6.78e-06 | 10.234 | 22.213 | 2.786 | 0.198 | At4g14160 | Sec23/Sec24 protein |
| Chr4 | 10318421 | 7.34e-06 | 9.879 | 19.987 | 3.134 | 0.189 | At4g18790 | Member of Nramp2 family |
| Chr4 | 5974306 | 8.12e-06 | 9.825 | 21.089 | 2.123 | 0.179 | At4g09430 | Disease resistance protein |
| Chr2 | 15805079 | 1.16e-05 | 9.614 | 19.435 | 3.101 | 0.162 | At2g37680 | Glucose-induced |
| Chr2 | 15810955 | 1.16e-05 | 9.614 | 19.435 | 3.101 | 0.162 | At2g37700 | Fatty acid hydroxylase |
| Chr4 | 9565257 | 1.56e-05 | 9.332 | 21.817 | -2.503 | 0.275 | At4g16990 | Resistance to leptosphaeria |
| Chr3 | 16044911 | 5.28e-05 | 8.173 | 19.896 | 2.827 | 0.096 | At3g44400 | Disease resistance protein |
| Chr3 | 1349568 | 5.71e-05 | 8.099 | 19.769 | 2.539 | 0.132 | At3g04900 | Heavy metal |
| Chr3 | 1388495 | 6.21e-05 | 8.019 | 21.335 | -2.996 | 0.150 | At3g05000 | Transport protein particle |
| Chr2 | 10822583 | 6.40e-05 | 7.990 | 19.629 | 2.939 | 0.138 | At2g25430 | Epsin n-terminal homology |
| Chr4 | 11468866 | 6.55e-05 | 7.969 | 19.796 | 2.682 | 0.120 | At4g21560 | Vacuolar protein sorting 28 |
| Chr3 | 2362006 | 7.11e-05 | 7.891 | 21.156 | -2.855 | 0.126 | At3g07380 | Glycosyltransferase family |
| Chr4 | 569954 | 7.29e-05 | 7.867 | 19.448 | 2.850 | 0.174 | At4g01380 | Plastocyanin-like domain- |
| Chr5 | 22901770 | 8.40e-05 | 7.733 | 19.830 | 2.674 | 0.114 | At5g56560 | Fbd, f-box and leucine rich |
| Chr2 | 2089769 | 5.31e-06 | 7.726 | 19.946 | 2.428 | 0.197 | At2g05635 | DNA repair DEAD helicase |
| Chr4 | 11453934 | 9.67e-05 | 7.600 | 19.832 | 2.664 | 0.114 | At4g21530 | Anaphase promoting |
| Chr5 | 18219139 | 1.02e-04 | 7.552 | 21.757 | -2.039 | 0.323 | At5g45100 | Boi-related gene 1 |
| Chr3 | 2365769 | 1.05e-04 | 7.520 | 20.901 | -3.513 | 0.066 | At3g07390 | Auxin-induced in root |
| Chr2 | 12902361 | 1.08e-04 | 7.494 | 19.717 | 3.012 | 0.120 | At2g30240 | Atchx13, cation/h+ |
| **Cumulative root length** | | | | | | | | |
| Chr1 | 23345414 | 1.88e-06 | 11.881 | 413.01 | 112.951 | 0.283 | At1g63005 | Microrna399b |
| Chr2 | 2089769 | 2.37e-06 | 11.756 | 415.94 | 80.539 | 0.257 | At2g05635 | DNA repair DEAD helicase |
| Chr3 | 1979512 | 2.42e-06 | 11.237 | 345.67 | 101.654 | 0.198 | At3g06450 | HCO3- transporter family |
| Chr5 | 2796339 | 2.88e-06 | 10.874 | 198.78 | 112.450 | 0.227 | At5g08620 | STRESS RESPONSE |
| Chr3 | 9878645 | 3.82e-06 | 10.678 | 561.46 | 211.494 | 0.054 | At3g26819 | Microrna169n |
| Chr5 | 18141321 | 9.74e-06 | 9.781 | 513.01 | 92.951 | 0.383 | At5g44930 | Arabinan deficient 2 |
| Chr5 | 18150355 | 1.37e-05 | 9.456 | 515.94 | 90.539 | 0.377 | At5g44940 | F-box/rni-like superfamily |
| Chr1 | 10446552 | 1.46e-05 | 9.393 | 401.10 | 147.970 | 0.084 | At1g29840 | Alpha/beta-hydrolases |
| Chr5 | 18673226 | 1.59e-05 | 9.313 | 122.91 | 94.613 | 0.323 | At5g46040 | Major facilitator |
| Chr5 | 21447074 | 1.83e-05 | 9.181 | 633.49 | -93.441 | 0.263 | At5g52890 | At hook motif-containing |
| Chr5 | 17566694 | 2.97e-05 | 8.717 | 631.64 | -92.015 | 0.257 | At5g43740 | Disease resistance protein |
| Chr3 | 23173163 | 3.33e-05 | 8.610 | 551.31 | 110.027 | 0.150 | At3g62660 | Galacturonosyltransferase- |
| Chr5 | 16002119 | 3.39e-05 | 8.592 | 552.60 | 120.119 | 0.132 | At5g39980 | Pigment-defective mutant3 |
| Chr5 | 19464368 | 3.59e-05 | 8.538 | 541.93 | 110.434 | 0.192 | At5g48020 | 2-oxoglutarate and fe |
| Chr5 | 18101364 | 3.64e-05 | 8.526 | 536.27 | 91.060 | 0.263 | At5g44830 | Pectin lyase-like |
| Chr1 | 23518484 | 4.60e-05 | 8.304 | 542.41 | 120.475 | 0.174 | At1g63420 | O-glucosyltransferase-like |
| Chr1 | 27760696 | 4.71e-05 | 8.280 | 566.30 | 166.553 | 0.054 | At1g73830 | Br enhanced expression 3 |
| Chr1 | 23340165 | 7.26e-05 | 7.871 | 452.51 | 139.188 | 0.084 | At1g62990 | Irregular xylem 11 |
| Chr1 | 23343168 | 7.26e-05 | 7.871 | 452.516 | 139.188 | 0.084 | At1g63000 | Udp-4-keto-6-deoxy-d-glucose-3,5-epimerase-4- |

| | | | | | | | | |
|---|---|---|---|---|---|---|---|---|
| Chr5 | 18138946 | 5.99e-05 | 8.053 | 665.02 | -81.253 | 0.497 | At5g44920 | Tir-kash protein involved in |
| **Root surface area** | | | | | | | | |
| Chr1 | 4244990 | 1.12e-06 | 11.971 | 51.456 | 12.567 | 0.179 | At1g12450 | SNARE associated Golgi |
| Chr4 | 9255532 | 1.33e-06 | 11.278 | 43.789 | 14.122 | 0.149 | At4g16380 | Heavy metal |
| Chr1 | 10439051 | 2.18e-06 | 11.123 | 47.491 | 12.556 | 0.098 | At1g29820 | Magnesium transporter |
| Chr3 | 3362581 | 2.88e-06 | 10.998 | 52.258 | 13.549 | 0.132 | At3g10740 | Encodes a bifunctional alpha-l- |
| Chr1 | 10446552 | 3.32e-06 | 10.810 | 46.978 | 13.508 | 0.084 | At1g29840 | Alpha/beta-hydrolases |
| Chr1 | 22814875 | 5.34e-06 | 10.356 | 47.221 | 14.074 | 0.072 | At1g61790 | Oligosaccharyltransferase |
| Chr4 | 18225877 | 5.69e-06 | 10.295 | 47.315 | 14.634 | 0.066 | At4g39120 | Histidine biosynthesis 7 |
| Chr1 | 23345414 | 6.44e-06 | 9.842 | 53.009 | 14.443 | 0.188 | At1g63005 | Microrna399b |
| Chr2 | 2089769 | 1.12e-05 | 9.833 | 45.213 | 13.234 | 0.145 | At2g05635 | DNA repair DEAD helicase |
| Chr1 | 10449946 | 1.16e-05 | 9.611 | 47.247 | 12.819 | 0.078 | At1g29850 | Programmed cell death |
| Chr5 | 17748147 | 2.10e-05 | 9.048 | 46.037 | 12.168 | 0.132 | At5g44090 | Calcium-binding ef-hand |
| Chr5 | 17749166 | 2.10e-05 | 9.048 | 46.037 | 12.168 | 0.132 | At5g44100 | Casein kinase i-like 7 |
| Chr5 | 17754825 | 2.10e-05 | 9.048 | 46.037 | 12.168 | 0.132 | At5g44110 | Atp-binding cassette a21 |
| Chr5 | 17756447 | 2.10e-05 | 9.048 | 46.037 | 12.168 | 0.132 | At5g44120 | Cra1 |
| Chr2 | 12126944 | 3.89e-05 | 8.463 | 44.243 | 8.187 | 0.305 | At2g28360 | Sit4 phosphatase-associated |
| Chr5 | 21447074 | 4.65e-05 | 8.292 | 53.210 | -7.528 | 0.263 | At5g52890 | At hook motif-containing |
| Chr1 | 23518484 | 6.24e-05 | 8.014 | 45.725 | 10.129 | 0.174 | At1g63420 | O-glucosyltransferase-like |
| Chr1 | 25516286 | 6.38e-05 | 7.994 | 52.106 | -8.853 | 0.162 | At1g68070 | Zinc finger, c3hc4 type ring |
| Chr1 | 25526656 | 6.81e-05 | 7.931 | 52.194 | -8.799 | 0.168 | At1g68120 | Basic pentacysteine protein |
| Chr5 | 14960677 | 7.32e-05 | 7.863 | 47.673 | 14.565 | 0.054 | At5g37660 | Plasmodesmata-located |
| **Root volume** | | | | | | | | |
| Chr2 | 8378956 | 2.35e-07 | 12.342 | 0.287 | 0.078 | 0.213 | At2g19360 | Protein of Unknown |
| Chr4 | 8014109 | 4.37e-07 | 12.114 | 0.332 | 0.118 | 0.354 | At4g13840 | HXXXD-type acyl- |
| Chr1 | 4509732 | 1.34e-06 | 11.889 | 0.306 | 0.079 | 0.099 | At1g13210 | autoinhibited Ca2+/ATPase |
| Chr1 | 23345414 | 1.69e-06 | 11.783 | 0.354 | 0.145 | 0.179 | At1g63005 | Microrna399b |
| Chr5 | 14960677 | 1.72e-06 | 11.773 | 0.336 | 0.153 | 0.054 | At5g37660 | Plasmodesmata-located |
| Chr4 | 18225877 | 1.76e-06 | 11.423 | 0.335 | 0.134 | 0.066 | At4g39120 | Histidine biosynthesis 7 |
| Chr4 | 8008049 | 2.78e-06 | 10.982 | 0.290 | 0.065 | 0.479 | At4g13820 | Leucine-rich repeat lrr |
| Chr2 | 2089769 | 3.45e-06 | 10.443 | 0.353 | 0.123 | 0.257 | At2g05635 | DNA repair DEAD helicase |
| Chr4 | 8014109 | 5.58e-06 | 10.314 | 0.376 | -0.100 | 0.120 | At4g13840 | Cer26 |
| Chr1 | 22814875 | 1.50e-05 | 9.367 | -0.131 | 0.118 | 0.072 | At1g61790 | Oligosaccharyltransferase |
| Chr2 | 5929527 | 1.93e-05 | 9.128 | 0.296 | 0.110 | 0.078 | At2g14080 | Disease resistance protein |
| Chr2 | 14269142 | 3.12e-05 | 8.672 | -0.042 | 0.102 | 0.084 | At2g33735 | Chaperone dnaj-domain |
| Chr1 | 22573729 | 3.23e-05 | 8.639 | 0.307 | 0.066 | 0.287 | At1g61215 | Bromodomain 4 |
| Chr1 | 20648069 | 3.40e-05 | 8.590 | 0.054 | 0.115 | 0.072 | At1g55325 | Mediator of rna polymerase |
| Chr4 | 18471932 | 3.55e-05 | 8.548 | -0.098 | 0.089 | 0.156 | At4g39800 | Inositol 3-phosphate |
| Chr4 | 18479378 | 3.55e-05 | 8.548 | -0.098 | 0.089 | 0.156 | At4g39830 | Cupredoxin superfamily |
| Chr4 | 18485513 | 3.55e-05 | 8.548 | -0.098 | 0.089 | 0.156 | At4g39838 | Potential natural antisense gene, locus overlaps with |
| Chr4 | 18486544 | 3.55e-05 | 8.548 | -0.098 | 0.089 | 0.156 | At4g39840 | Cell wall integrity/stress response component-like |

| | | | | | | | | |
|---|---|---|---|---|---|---|---|---|
| Chr4 | 18489909 | 3.55e-05 | 8.548 | -0.098 | 0.089 | 0.156 | At4g39850 | Atp-binding cassette d1 |
| Chr1 | 21951156 | 3.67e-05 | 8.518 | 0.326 | 0.093 | 0.138 | At1g59725 | Dnaj heat shock family |
| **Average root diameter** | | | | | | | | |
| Chr1 | 23642153 | 2.33e-07 | 12.453 | 0.268 | 0.045 | 0.315 | At1g63730 | Disease resistance protein |
| Chr5 | 2800515 | 2.78e-07 | 12.233 | 0.277 | 0.038 | 0.230 | At5g08640 | DDT domain-containing |
| Chr2 | 12001039 | 3.67e-07 | 12.176 | 0.297 | 0.027 | 0.198 | At2g28150 | Protein of unknown |
| Chr5 | 16040827 | 4.01e-07 | 12.089 | 0.212 | 0.049 | 0.130 | At5g40080 | Mitochondrial ribosomal |
| Chr2 | 2089769 | 4.31e-07 | 11.994 | 0.314 | 0.049 | 0.369 | At2g05635 | DNA repair DEAD helicase |
| Chr4 | 8014109 | 1.02e-06 | 11.941 | 0.287 | -0.036 | 0.120 | At4g13840 | Cer26 |
| Chr4 | 8001922 | 5.04e-06 | 10.411 | 0.089 | -0.024 | 0.293 | At4g13800 | Magnesium transporter |
| Chr3 | 19114519 | 8.12e-06 | 9.955 | 0.272 | 0.050 | 0.054 | At3g51530 | F-box/rni-like/fbd-like |
| Chr3 | 19117045 | 8.12e-06 | 9.955 | 0.272 | 0.050 | 0.054 | At3g51540 | Mucin-5ac-like protein |
| Chr1 | 23345414 | 9.32e-06 | 9.883 | 0.263 | 0.043 | 0.313 | At1g63005 | Microrna399b |
| Chr3 | 19575910 | 1.05e-05 | 9.708 | 0.261 | 0.025 | 0.347 | At3g52820 | Purple acid phosphatase 22 |
| Chr3 | 19669649 | 2.02e-05 | 9.085 | 0.270 | 0.034 | 0.114 | At3g53050 | D-galactoside/l-rhamnose |
| Chr5 | 4283945 | 2.35e-05 | 8.940 | 0.272 | 0.040 | 0.078 | At5g13360 | Auxin-responsive gh3 |
| Chr5 | 4288362 | 2.35e-05 | 8.940 | 0.272 | 0.040 | 0.078 | At5g13370 | Auxin-responsive gh3 |
| Chr5 | 4290313 | 2.35e-05 | 8.940 | 0.272 | 0.040 | 0.078 | At5g13380 | Auxin-responsive gh3 |
| Chr5 | 4299175 | 2.35e-05 | 8.940 | 0.272 | 0.040 | 0.078 | At5g13400 | Major facilitator |
| Chr5 | 4299756 | 2.35e-05 | 8.940 | 0.272 | 0.040 | 0.078 | At5g13410 | Fkbp-like peptidyl-prolyl |
| Chr3 | 7949384 | 2.60e-05 | 8.847 | 0.296 | -0.022 | 0.413 | At3g22421 | F-box/associated interaction |
| Chr5 | 4281420 | 2.98e-05 | 8.716 | 0.271 | 0.037 | 0.090 | At5g13350 | Auxin-responsive gh3 |
| Chr3 | 14680488 | 3.73e-05 | 8.502 | 0.272 | 0.035 | 0.084 | At3g42560 | Pentatricopeptide repeat |

*TestStatt- calculate F-type test statistics for a general linear hypothesis for each of G genes. * Beta0 and Beta1 are resulting coefficient from a fit and SE *MFA- minor allele frequency

**Table S4.** Primers used for genotyping and RT-qPCR analyses in this study.

| Locus | T-DNA mutants* | Forward | Reverse |
|---|---|---|---|
| *At1g63005* | Salk_151454 | TGGGAGGTCATCCTACCAAG | AGGCGCCCTAAAACTAGTGAG |
| *At2g05635* | Salk_074272 | CCTGTCATCATCATGCAACTC | TCAGTTTTTATGGTAAAGTTTAATTGG |
| Border primer | LBb1.3 | ATTTTGCCGATTTCGGAAC | |
| **Primers for gene expression studies** | | | |
| *At1g63005* | RT-qPCR | AGGGCGCCTCTCCATTG | TCGATCATCGGAAATTTTCGA |
| *At2g05635* | RT-qPCR | TTCGTGAGTATAGGAAAACTGGTTACA | CATGAACACCTAATCCTCCTACCAA |
| *Actin8* | RT-qPCR | CCCAAAAGCCAACAGAGAGA | CATCACCAGAGTCCAACACAAT |

*-claimed to be homozygous T-DNA mutants from ABRC (http://abrc.osu.edu).
